# Facile access to 3-sulfonylquinolines via Knoevenagel condensation/aza-Wittig reaction cascade involving *ortho*-azidobenzaldehydes and β-ketosulfonamides and sulfones

**DOI:** 10.3762/bjoc.19.60

**Published:** 2023-06-09

**Authors:** Ksenia Malkova, Andrey Bubyrev, Stanislav Kalinin, Dmitry Dar’in

**Affiliations:** 1 Saint Petersburg State University, Saint Petersburg 199034, Russian Federation

**Keywords:** aza-Wittig reaction, azides, cyclocondensation, quinolones, sulfonamides

## Abstract

Quinoline-based sulfonyl derivatives, and especially sulfonamides, are relevant and promising structures for drug design. We have developed a new convenient protocol for the synthesis of 3-sulfonyl-substituted quinolines (sulfonamides and sulfones). The approach is based on a Knoevenagel condensation/aza-Wittig reaction cascade involving *o*-azidobenzaldehydes and ketosulfonamides or ketosulfones as key building blocks. The protocol is appropriate for both ketosulfonyl reagents and α-sulfonyl-substituted alkyl acetates providing the target quinoline derivatives in good to excellent yields.

## Introduction

**Figure 1:**
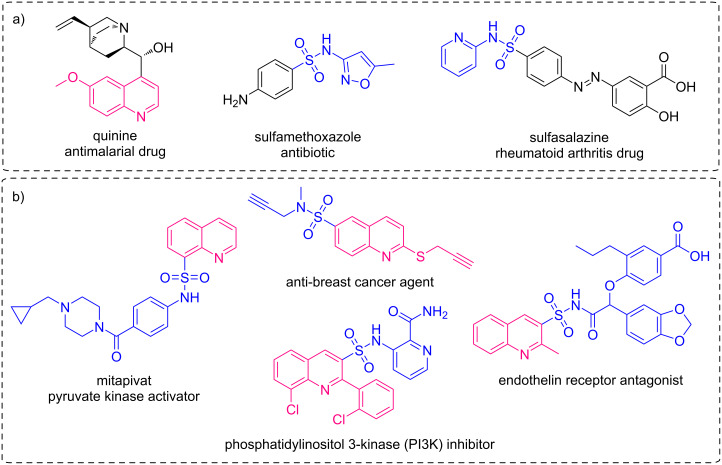
a) Conventional drugs containing either a sulfonamide fragment or a quinoline core; b) biologically active quinoline sulfonamides.

The quinoline scaffold has a wide occurrence among natural products [1] and is a key structural component of several pharmaceuticals, agrochemicals, dyestuffs, and materials. Particularly, the well-known antimalarial alkaloid quinine isolated from *Cinchona* bark comprises a quinoline core (Figure 1a) [2]. Moreover, numerous quinoline derivatives have been recently reported to possess intriguing pharmacological activities [3] including antiprotozoal [4–7], antitubercular [8–9], anticancer [10–11], anti-inflammatory [12], antioxidant [13], anti-HIV [14], antifungal [15], and an antineurodegenerative effect [16]. Hence, designing novel quinoline construction and functionalization techniques resulting in new or rare derivatives [17–26] is an important mission in the field of drug discovery and medicinal chemistry.

The sulfonamide group is a known privileged motif in drug design often serving as a linker or pharmacophore group. In fact, more than one hundred FDA-approved drugs are sulfonamide-bearing small molecules. Screening libraries of aromatic and heteroaromatic sulfonamides gave rise to the discovery of multiple physiologically active compounds [27–30] including important pharmaceuticals, such as sulfamethoxazole and sulfasalazine (Figure 1a). In this context, combining sulfonamide and quinoline fragments promises to be a fruitful strategy to identify diverse types of therapeutically relevant compounds. The effectiveness of this approach is demonstrated by a series of bioactive structures developed recently, and, significantly, quinoline-3-sulfonamides are frequently encountered among such pharmacologically active hybrids (Figure 1b) [31–34].

Despite these facts, the diversity of quinoline-3-sulfonamides reported in the literature is limited due to obstacles in the synthesis of quinoline-3-sulfonyl chlorides which are the most common reagents for their preparation. As a possible solution, the approach to the heterocyclic core construction from a sulfonamide-containing building block may be considered. In turn, diversely substituted quinoline-3-sulfones are available through a range of synthetic methodologies suggested recently. Cyclization strategies [35–45] as well as cycloaddition/cyclocondensation techniques [46–51] represent those with hetero-ring construction. Alternative approaches rely on a peripheral modification of various substrates, such as 3-bromoquinolines [52–55], quinoline-3-boronic acids [56], and diazonium salts [57].

**Figure 2:**
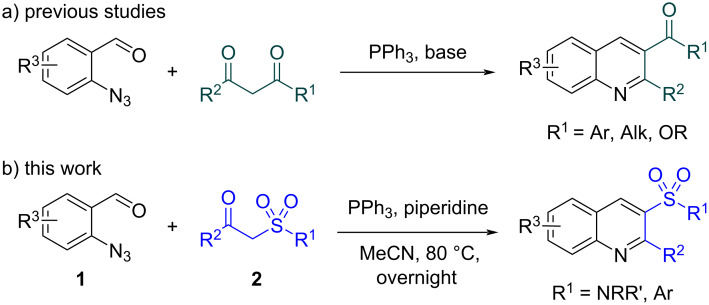
Knoevenagel condensation/aza-Wittig reaction cascade for the quinoline core formation.

When considering general methods for the quinoline core formation, aromatic *ortho*-substituted carbonyl compounds attract attention as decent and easily available reagents. While the *ortho*-amino carbonyl reagents are not always easily accessible and sometimes unstable (e.g., aminoaldehydes), both *o*-azidoaldehydes [58–65] and *o*-azidoketones [66–69] have been proved to be appropriate substrates for quinoline derivatives synthesis. Recently, the method for the synthesis of 3-acyl-substituted quinolines from *o*-azidobenzaldehydes and 1,3-dicarbonyl compounds was reported [70–71] (Figure 2a). A combination of Knoevenagel condensation and aza-Wittig reaction allowed to build up target products in high yields. In case of [70], the procedure was predominantly applied for the preparation of the corresponding esters.

Inspired by this study, we became interested to utilize *o*-azidobenzaldehydes **1** in combination with ketosulfonamides/ketosulfones **2** as precursors in a new convenient synthetic procedure leading towards 3-sulfonyl-substituted quinolines (sulfonamides and sulfones) (Figure 2b). Herein, we report the successful implementation of this approach.

## Results and Discussion

**Scheme 1:**
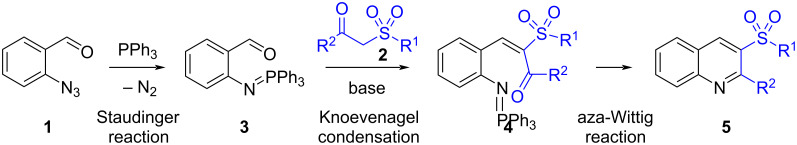
Key reaction steps during the synthesis of 3-sulfonyl-substituted quinolines.

The Knoevenagel condensation/aza-Wittig reaction cascade was used for the preparation of 3-sulfonyl-substituted quinolines. The process proceeds in a domino fashion including the following steps: the formation of iminophosphorane **3** from *o*-azidobenzaldehyde (**1**) and PPh_3_ followed by the base-mediated Knoevenagel condensation results in compound **4**; a subsequent intramolecular aza-Wittig reaction leads to the desired product **5** (Scheme 1).

Starting from the reaction conditions reported previously, we began our investigation using *o*-azidobenzaldehyde (**1a**), 2-oxopropanesulfonamide **2a**, triphenylphosphine, and diethylamine as reagents for the quinoline-3-sulfonamide assembly (Table 1). The reaction mixture was stirred in MeCN at 95 °C for 6 h which led to a mediocre yield of the target compound **5a** estimated by NMR (Table 1, entry 1). Different organic bases were tested, with piperidine performing most efficiently (Table 1, entry 4). Next it was found out that using *o*-azidobenzaldehyde (**1a**), PPh_3_, and an excess of piperidine in relation to ketosulfonamide **2a** resulted in higher yields of quinoline **5a**. The optimal solvent volume (the concentration of **2a**) was chosen considering both reaction yields and practical reasons. Subsequent tuning of temperature and reaction time ensured quantitative NMR yield in the model reaction (Table 1, entry 13).

**Table 1:** Optimization of reaction conditions.^a^

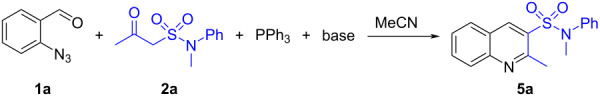							

Entry	Base (equiv)	**1a**, equiv	**2a**, equiv	PPh_3_, equiv	*c***_2a_**, M	Δ, °C	NMR yield, %

1	Et_2_NH (1.0)	1.0	1.2	1.2	0.147	95	31
2	Et_3_N (1.0)	1.0	1.2	1.2	0.147	95	43
3	pyrrolidine (1.0)	1.0	1.2	1.2	0.147	95	45
4	piperidine (1.0)	1.0	1.2	1.2	0.147	95	63
5	piperidine (1.0)	1.0	1.0	1.2	0.147	95	63
6	piperidine (1.1)	1.1	1.0	1.3	0.147	95	69
7	piperidine (1.25)	1.25	1.0	1.5	0.147	95	82
8	piperidine (0.5)	1.25	1.0	1.5	0.147	95	73
9	piperidine (1.5)	1.25	1.0	1.5	0.147	95	75
10	piperidine (1.25)	1.25	1.0	1.5	0.294	95	58
11	piperidine (1.25)	1.25	1.0	1.5	0.074	95	91
12	piperidine (1.25)	1.25	1.0	1.5	0.037	95	95
13	piperidine (1.25)	1.25	1.0	1.5	0.074	80	99^b^
14	piperidine (1.25)	1.25	1.0	1.5	0.074	65	91

^a^Reaction scale ‒ 0.1 mmol, reaction time ‒ 6 h. ^b^Reaction was run overnight (16 h).

**Scheme 2:**
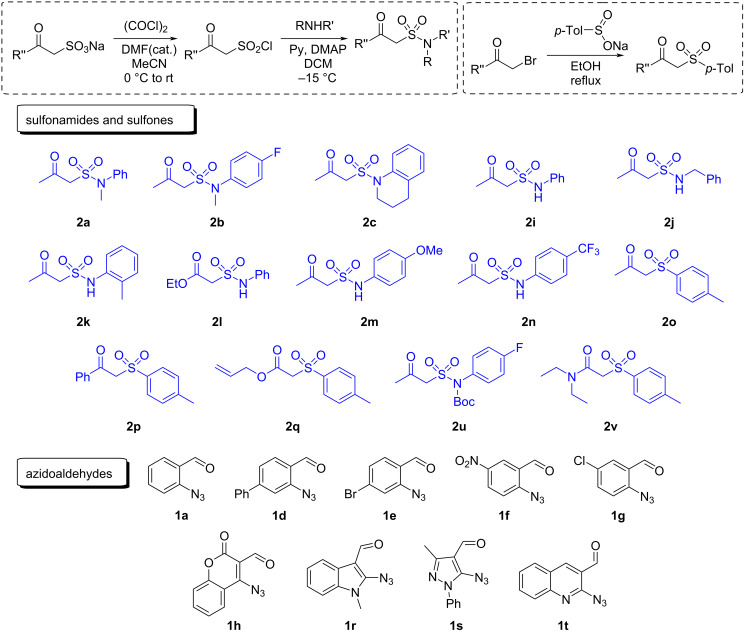
Synthetic routes to sulfonamides and sulfones **2** and the set of reagents for the preparation of compounds **5**.

A remarkable advantage of the approach devised is that all starting materials including sulfonyl compounds are easily accessible. Furthermore, the preparation techniques are flexible concerning the variations of substituents, which is of high importance for the potential medicinal chemistry applications. Scheme 2 illustrates unobstructed synthetic routes [72–74] to sulfonamides and sulfones **2**, and the diversity of reagents used to prepare the target products **5**.

With the reaction conditions optimized, a series of novel tertiary quinoline-3-sulfonamides and quinoline-3-sulfones was successfully generated. In case of the secondary quinoline-3-sulfonamide synthesis, the increase of reagent excesses in relation to ketosulfonamide resulted in the conversion and an increase of the yield as observed by TLC (see GP2 in Supporting Information File 1).

**Scheme 3:**
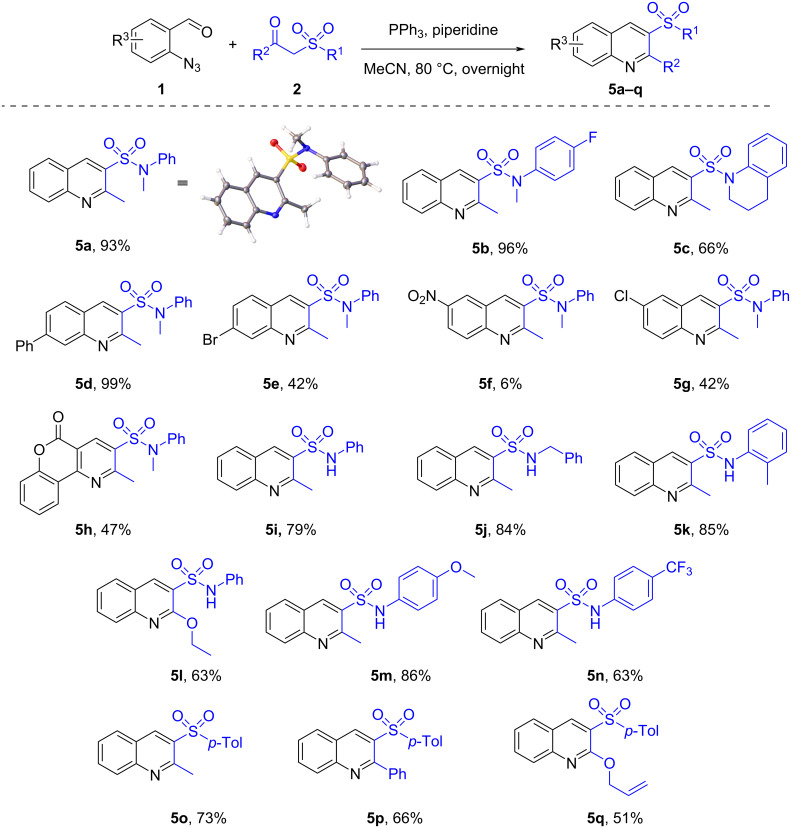
Preparation of 3-sulfonyl substituted quinolines **5a–q**.

Chromatographic purification afforded compounds **5a–q** mostly in good to excellent yields (Scheme 3). The product structures were confirmed by the standard set of characterization data as well as the single-crystal X-ray structure of the representative compound **5a**.

The presence of an electron-withdrawing group in the *o*-azidobenzaldehyde leads to decreased yields of target products (Scheme 3, **5e** and **5g**). The drop was especially dramatic for the nitro group containing reagent **1f**. Indeed, the transformation was accompanied by a number of side reactions according to TLC. To our delight, the protocol turned out to be suitable for α-sulfonyl-substituted alkyl acetates leading to 2-alkoxyquinolines. Compounds **5l** an **5q** were obtained in 63 and 51% yield, respectively (Scheme 3). It is worth noticing that chromenopyridine-3-sulfonamide **5h** derived from heterocyclic azidoaldehyde **1h** was also successfully synthesized following the methodology designed.

**Scheme 4:**
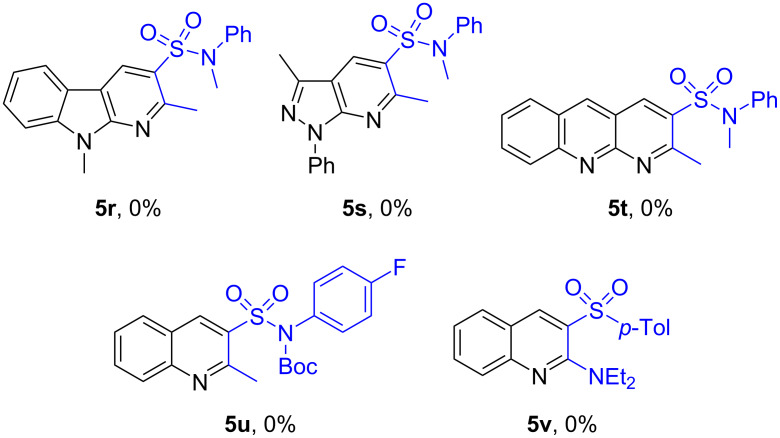
3-Sulfonyl-substituted quinolines **5r–v** that failed to be synthesized.

Some limitations on the substrate scope for the protocol proposed were found out during the course of the study. Indole- and pyrazole-based azidoaldehydes **1r** and **1s** failed to provide the desired compounds **5r** and **5s** (Scheme 4). The reaction stopped on the iminophosphorane formation and did not progress further likely due to carbonyl group deactivation. Furthermore, while implementing the protocol for 2-azidoquinoline-3-carbaldehyde (**1t**), a low conversion of this reagent was detected, which can be explained by the fact that **1t** tends to exist in the inactive tetrazole form. In addition, our attempt to involve Boc-protected ketosulfonamide **2u** in the transformation resulted in the *N*-deprotected product. Finally, *N,N*-diethyl-2-tosylacetamide (**2v**) appeared to be incapable of entering the Knoevenagel condensation in the suggested conditions.

## Conclusion

In summary, we have successfully developed a new straightforward protocol for the synthesis of 3-sulfonyl-substituted quinolines (sulfonamides and sulfones). The approach is based on a Knoevenagel condensation/aza-Wittig reaction cascade for the quinoline core assembly. Hence, *o*-azidobenzaldehyde, ketosulfonamide or ketosulfone were utilized as key building blocks. The method devised proved to be a convenient approach to the preparation of 3-sulfonyl-substituted quinolines. The desired compounds were obtained in good to excellent yields. Importantly, the protocol was found suitable not only for ketosulfonyl reagents but also for α-sulfonyl-substituted alkyl acetates providing a pathway to 2-alkoxyquinolines.

## Supporting Information

Deposition number 2242072 (for **5a**) contain the supplementary crystallographic data for this paper. These data are provided free of charge by the joint Cambridge Crystallographic Data Centre and Fachinformationszentrum Karlsruhe Access Structures service https://www.ccdc.cam.ac.uk/structures.

File 1 General experimental information, X-ray crystallographic data, synthetic procedures, analytical data and NMR spectra for the reported compounds.
