# Supplementary material for: Facile access to 3-sulfonylquinolines via Knoevenagel condensation/aza-Wittig reaction cascade involving ortho-azidobenzaldehydes and β-ketosulfonamides and sulfones
Source: Beilstein J Org Chem. 2023 Jun 9;19:800–7. doi: 10.3762/bjoc.19.60 (PMC10280061; doi:10.3762/bjoc.19.60)
Supplement: File 1 — General experimental information, X-ray crystallographic data, synthetic procedures, analytical data and NMR spectra for the reported compounds. [file Beilstein_J_Org_Chem-19-800-s001.pdf]

BEILSTEIN JOURNAL OF ORGANIC CHEMISTRY

Supporting Information

for

Facile access to 3-sulfonylquinolines via Knoevenagel condensation/aza-Wittig reaction cascade involving *ortho*-azidobenzaldehydes and β-ketosulfonamides and sulfones

Ksenia Malkova, Andrey Bubyrev, Stanislav Kalinin and Dmitry Dar'in

Beilstein J. Org. Chem. **2023,** *19,* 800–807. doi:10.3762/bjoc.19.60

General experimental information, X-ray crystallographic data, synthetic procedures, analytical data and NMR spectra for the reported compounds

License and Terms: This is a supporting information file under the terms of the Creative Commons Attribution License (https://creativecommons.org/licenses/by/4.0). Please note that the reuse, redistribution and reproduction in particular requires that the author(s) and source are credited and that individual graphics may be subject to special legal provisions.
The license is subject to the *Beilstein Journal of Organic Chemistry* terms and conditions: (https://www.beilstein-journals.org/bjoc/terms)

Table of contents

I. Materials and methods S2

II. The synthesis of starting materials S3

III. NMR optimization of reaction conditions S6

*IV. General procedures for the synthesis of **5a–q*** S7

V. NMR spectra S12

VI. X-ray crystallographic data S31

VII. References S32

I. Materials and Methods

All commercial reagents were used without purification. NMR spectra were recorded using a Bruker Avance III spectrometer in $CDCl_3$ or DMSO-d_6 (^{1}H: 400.13 MHz, ^{13}C: 100.61 MHz and 125.73 MHz, ^{19}F: 376.50 MHz). All chemical shifts are reported in parts per million (ppm). The residual solvent peak was used as internal standard: $CDCl_3$ (7.26 for ^{1}H and 77.16 ppm for ^{13}C), DMSO-d_6 (2.50 for ^{1}H and 39.52 ppm for ^{13}C). Standard abbreviations were used in the description of resonances. Coupling constants (*J*) are quoted to the nearest 0.1 Hz. Mass spectra were recorded with a HRMS-ESI-qTOF spectrometer (electrospray ionization mode). Melting points were determined with a melting point apparatus Stuart SMP10 in open capillary tubes. Single crystal X-ray data were obtained using an Agilent Technologies SuperNova Atlas diffractometer at a temperature of 100 K. Analytical thin layer chromatography was carried out on UV-254 silica gel plates using appropriate eluents. Compounds were visualized with short wavelength UV light. Column chromatography was performed using silica gel Merk grade 60 (0.040−0.063 mm) 230−400 mesh.

II. The synthesis of starting materials

*Azidoaldehydes **1***

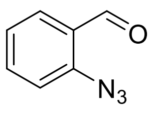

1a

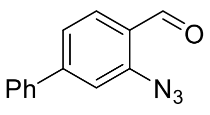

1d

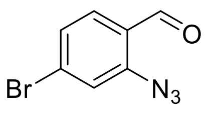

1e

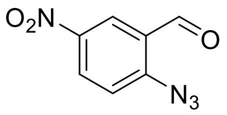

1f

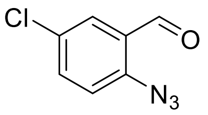

1g

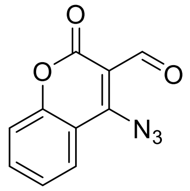

1h

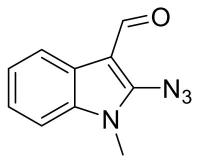

1r

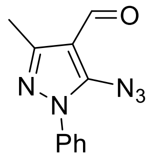

1s

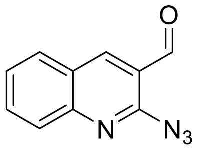

1t

Azidoaldehydes **1a**,**d**–**g**,**r**,**t** were synthesized as described previously [1].

4-Azido-2-oxo-2*H*-chromene-3-carbaldehyde (**1h**) was prepared from 4-chloro-2-oxo-2*H*-chromene-3-carbaldehyde according to a literature technique [2].

5-Azido-3-methyl-1-phenyl-1*H*-pyrazole-4-carboxaldehyde (**1s**) was synthesized from 5-chloro-3-methyl-1-phenyl-1*H*-pyrazole-4-carbaldehyde as described in the literature [3].

*Ketosulfonamides and ketosulfones **2***

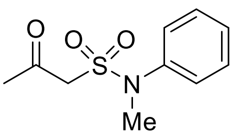

2a

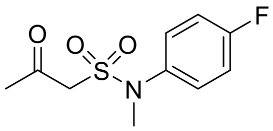

2b

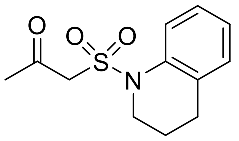

2c

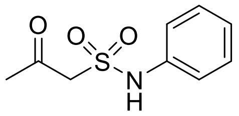

2i

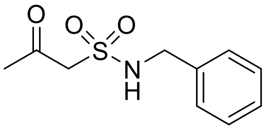

2j

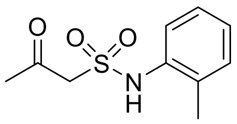

2k

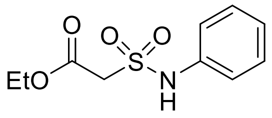

2l

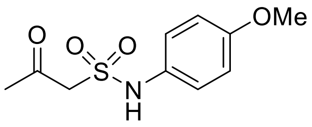

2m

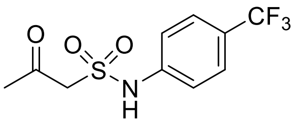

2n

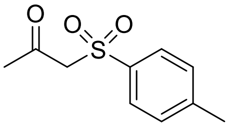

2o

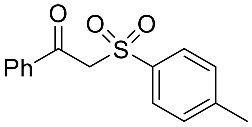

2p

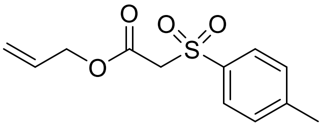

2q

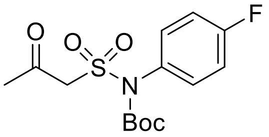

2u

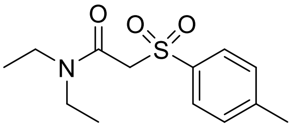

2v

Ketosulfonamides **2a–c**,**i–n** were synthesized as described previously [4,5].

Reagents **2o–q** were prepared according to a literature technique [6].

tert-Butyl (4-fluorophenyl)((2-oxopropyl)sulfonyl)carbamate (**2u**) was prepared from *N*-(4-fluorophenyl)-2-oxopropane-1-sulfonamide by reaction with the anhydride Boc_2O in the presence of DMAP according to the literature [7].

N,*N*-Diethyl-2-tosylacetamide (**2v**) was synthesized as described in the literature [8].

*N-Methyl-2-oxo-N-phenylpropane-1-sulfonamide (**2a**)*

^{1}H NMR (400 MHz, $CDCl_3$) δ 7.45 – 7.35 (m, 4H), 7.33 – 7.28 (m, 1H), 4.00 (s, 2H), 3.32 (s, 3H), 2.36 (s, 3H). ^{13}C{^{1}H} NMR (101 MHz, $CDCl_3$) δ 197.6, 140.6, 129.5, 128.0, 127.3, 60.8, 39.4, 31.3. HRMS (ESI) m/z: $[M+Na]^+$ Calcd for $C_{10}H_{13}NNaO_3S$ $[M+Na]^+$ 250.0508; Found 250.0503.

*N-(4-Fluorophenyl)-N-methyl-2-oxopropane-1-sulfonamide (**2b**)*

^{1}H NMR (400 MHz, $CDCl_3$) δ 7.47 – 7.38 (m, 2H), 7.13 – 7.03 (m, 2H), 4.00 (s, 2H), 3.31 (s, 3H), 2.40 (s, 3H). ^{13}C{^{1}H} NMR (101 MHz, $CDCl_3$) δ 197.8, 162.0 (d, C-F, $^1J_{C-F}$ = 248.6 Hz), 136.6 (d, C-F, $^4J_{C-F}$ = 3.2 Hz), 129.6 (d, C-F, $^3J_{C-F}$ = 8.8 Hz), 116.5 (d, C-F, $^2J_{C-F}$ = 22.7 Hz), 60.9, 39.9, 31.5. ^{19}F{^{1}H} NMR (376 MHz, $CDCl_3$) δ -112.9. HRMS (ESI) m/z: $[M+Na]^+$ Calcd for $C_{10}H_{12}FNNaO_3S$ $[M+Na]^+$ 268.0414; Found 268.0413.

*1-((3,4-Dihydroquinolin-1(2H)-yl)sulfonyl)propan-2-one (**2c**)*

^{1}H NMR (400 MHz, $CDCl_3$) δ 7.66 (dd, *J* = 8.2, 1.1 Hz, 1H), 7.21 – 7.03 (m, 3H), 4.04 (s, 2H), 3.86 – 3.74 (m, 2H), 2.84 (t, *J* = 6.6 Hz, 2H), 2.35 (s, 3H), 2.01 (p, *J* = 6.6 Hz, 2H). ^{13}C{^{1}H} NMR (101 MHz, $CDCl_3$) δ 196.5, 136.3, 130.0, 129.9, 127.0, 125.1, 122.5, 62.9, 47.2, 31.1, 27.1, 22.7. HRMS (ESI) m/z: $[M+Na]^+$ Calcd for $C_{12}H_{15}NNaO_3S$ $[M+Na]^+$ 276.0665; Found 276.0659.

*2-Oxo-N-(o-tolyl)propane-1-sulfonamide (**2k**)*

^{1}H NMR (400 MHz, $CDCl_3$) δ 7.45 (dd, *J* = 7.8, 1.5 Hz, 1H), 7.25 – 7.12 (m, 3H), 6.66 (s, 1H), 4.13 (s, 2H), 2.36 (s, 6H). ^{13}C{^{1}H} NMR (101 MHz, $CDCl_3$) δ 197.9, 134.3, 132.3, 131.6, 127.3, 126.8, 123.2, 61.0, 31.6, 18.1. HRMS (ESI) m/z: $[M-H]^-$ Calcd for $C_{10}H_{12}NO_3S$ $[M-H]^-$ 226.0543; Found 226.0550.

*2-Oxo-N-(4-(trifluoromethyl)phenyl)propane-1-sulfonamide (**2n**)*

^{1}H NMR (400 MHz, $CDCl_3$) δ 7.62 (d, *J* = 8.2 Hz, 2H), 7.40 (d, *J* = 8.2 Hz, 2H), 7.22 (s, 1H), 4.09 (s, 2H), 2.36 (s, 3H). ^{13}C{^{1}H} NMR (101 MHz, $CDCl_3$) δ 198.1, 139.5, 128.2 (q, C-F, $^2J_{C-F}$ = 33.1 Hz), 127.1 (q, C-F, $^3J_{C-F}$ = 3.7 Hz), 123.9 (q, C-F, $^1J_{C-F}$ = 271.6 Hz), 121.5, 59.9, 31.8. ^{19}F{^{1}H} NMR (376 MHz, $CDCl_3$) δ -62.4. HRMS (ESI) m/z: $[M-H]^-$ Calcd for $C_{10}H_9F_3NO_3S$ $[M-H]^-$ 280.0261; Found 280.0266.

*Allyl 2-tosylacetate (**2q**)*

^{1}H NMR (400 MHz, $CDCl_3$) δ 7.66 (dd, *J* = 8.2, 1.1 Hz, 1H), 7.21 – 7.03 (m, 3H), 4.04 (s, 2H), 3.86 – 3.74 (m, 2H), 2.84 (t, *J* = 6.6 Hz, 2H), 2.35 (s, 3H), 2.01 (p, *J* = 6.6 Hz, 2H). $^{13}C\{^{1}H\}$ NMR (101 MHz, $CDCl_3$) δ 196.5, 136.3, 130.0, 129.9, 127.0, 125.1, 122.5, 62.9, 47.2, 31.1, 27.1, 22.7. HRMS (ESI) m/z: $[M+Na]^+$ Calcd for $C_{12}H_{14}NaO_4S$ $[M+Na]^+$ 277.0505; Found 277.0507.

III. NMR optimization of reaction conditions

A mixture of 2-azidobenzaldehyde (**1a**), *N*-methyl-2-oxo-*N*-phenylpropane-1-sulfonamide (**2a**), and PPh_3 in MeCN was stirred for a few minutes. To the resulting solution a base was added. The solution was stirred for 6 h/overnight. A small portion of the solution was transported into a flask and evaporated in vacuo. The standard solution of quinoline in $CDCl_3$ was added to the oily residue. The resulting solution was transferred into an NMR tube.

IV. General procedures for the synthesis of 5a–q

General procedure for the synthesis of quinoline-3-sulfonamides 5a–g, chromenopyridine-3-sulfonamide 5h and quinoline-3-sulfones 5o–q (GP1)

A mixture of azidoaldehyde **1** (0.625 mmol, 1.25 equiv), ketosulfonamide or ketosulfone **2** (0.5 mmol, 1 equiv), and PPh_3 (197 mg, 0.75 mmol, 1.5 equiv) in 6.5 mL of MeCN was stirred for a few minutes. To the resulting solution piperidine (61.7 µL, 0.625 mmol, 1.25 equiv) was added. The solution was stirred at 80 °C overnight. The mixture was adsorbed onto silica and chromatographed with a hexane/ethyl acetate system using gradient elution to give desired product **5a–g** or **5o,q**.

In case of **5h**, a mixture of azidoaldehyde **1h** (67 mg, 0.313 mmol, 1.25 equiv), ketosulfonamide **2a** (57 mg, 0.25 mmol, 1 equiv), and PPh_3 (98 mg, 0.375 mmol, 1.5 equiv) in 3.25 ml of MeCN was stirred for a few minutes. To the resulting solution piperidine (30.9 µL, 0.313 mmol, 1.25 equiv) was added. Further operations were carried out as described above.

In case of **5p**, after stirring overnight solvent was removed in vacuo. The crude solid product was recrystallized from hexane/benzene/DCM (2:2:3, 7 mL) system to give pure compound **5p**.

General procedure for the synthesis of quinoline-3-sulfonamides 5i–n (GP2)

A mixture of azidoaldehyde **1** (0.625 mmol, 1.56 equiv), ketosulfonamide **2** (0.4 mmol, 1 equiv), and PPh_3 (197 mg, 0.75 mmol, 1.88 equiv) in 6.5 ml of MeCN was stirred for a few minutes. To the resulting solution piperidine (61.7 µL, 0.625 mmol, 1.56 equiv) was added. The solution was stirred at 80 °C overnight. The mixture was adsorbed onto silica and chromatographed with a hexane/ethyl acetate system using gradient elution to give desired product **5i–n**.

*N,2-Dimethyl-N-phenylquinoline-3-sulfonamide (**5a**)*

Prepared from 2-azidobenzaldehyde (**1a**) and *N*-methyl-2-oxo-*N*-phenylpropane-1-sulfonamide **2a** according to **GP1**. Eluent: hexane/ethyl acetate from 100:0 to 75:25. Yield: 145 mg (93%). Light yellow powder; mp 110-111°C. ^{1}H NMR (400 MHz, $CDCl_3$) δ 8.65 (s, 1H), 8.04 (dd, *J* = 8.8, 1.2 Hz, 1H), 7.90 – 7.79 (m, 2H), 7.58 (ddd, *J* = 8.2, 7.1, 1.2 Hz, 1H), 7.35 – 7.23 (m, 3H), 7.22 – 7.17 (m, 2H), 3.31 (s, 3H), 2.70 (s, 3H). ^{13}C{^{1}H} NMR (101 MHz, $CDCl_3$) δ 155.9, 148.8, 141.1, 140.3, 132.6, 130.6, 129.4, 128.8, 128.7, 127.9, 127.4, 127.0, 125.4, 38.8, 24.7. HRMS (ESI) m/z: $[M+Na]^+$ Calcd for $C_{17}H_{16}N_2NaO_2S$ $[M+Na]^+$ 335.0825; Found 335.0825.

*N-(4-Fluorophenyl)-N,2-dimethylquinoline-3-sulfonamide (**5b**)*

Prepared from 2-azidobenzaldehyde (**1a**) and *N*-(4-fluorophenyl)-*N*-methyl-2-oxopropane-1-sulfonamide (**2b**) according to **GP1**. Eluent: hexane/ethyl acetate from 100:0 to 75:25. Yield: 159 mg (96%). Light yellow powder; mp 93-94°C. ^{1}H NMR (400 MHz, $CDCl_3$) δ 8.63 (s, 1H), 8.11 – 8.01 (m, 1H), 7.87 – 7.81 (m, 2H), 7.59 (ddd, *J* = 8.2, 6.9, 1.2 Hz, 1H), 7.20 – 7.13 (m, 2H), 7.02 – 6.94 (m, 2H), 3.28 (s, 3H), 2.75 (s, 3H). ^{13}C{^{1}H} NMR (101 MHz, $CDCl_3$) δ 161.9 (d, C-F, $^1J_{C-F}$ = 248.6 Hz), 155.8, 148.9, 140.5, 137.0 (d, $^4J_{C-F}$ = 3.2 Hz), 132.7, 130.4, 129.2 (d, C-F, $^3J_{C-F}$ = 8.6 Hz), 128.8, 128.7, 127.5, 125.4, 116.4 (d, C-F, $^2J_{C-F}$ = 22.8 Hz), 39.1, 24.8. ^{19}F{^{1}H} NMR (376 MHz, $CDCl_3$) δ

-112.9. HRMS (ESI) m/z: $[M+Na]^+$ Calcd for $C_{17}H_{15}FN_2NaO_2S$ $[M+Na]^+$ 353.0730; Found 353.0726.

*3-((3,4-Dihydroquinolin-1(2H)-yl)sulfonyl)-2-methylquinoline (**5c**)*

Prepared from 2-azidobenzaldehyde (**1a**) and 1-((3,4-dihydroquinolin-1(2*H*)-yl)sulfonyl)propan-2-one (**2c**) according to **GP1**. Eluent: hexane/ethyl acetate from 100:0 to 75:25. Yield: 111 mg (66%). Light orange oil. ^{1}H NMR (400 MHz, $CDCl_3$) δ 8.75 (s, 1H), 8.08 – 8.03 (m, 1H), 7.88 (dd, *J* = 8.2, 1.5 Hz, 1H), 7.84 (ddd, *J* = 8.2, 6.9, 1.5 Hz, 1H), 7.61 (ddd, *J* = 8.2, 6.9, 1.2 Hz, 1H), 7.48 – 7.44 (m, 1H), 7.15 – 7.04 (m, 3H), 3.87 – 3.81 (m, 2H), 2.77 (s, 3H), 2.70 (t, *J* = 6.8 Hz, 2H), 1.90 – 1.78 (m, 2H). ^{13}C{^{1}H} NMR (101 MHz, $CDCl_3$) δ 155.7, 148.7, 139.3, 137.2, 133.4, 132.6, 130.4, 129.6, 128.9, 128.8, 127.5, 126.7, 125.5, 125.2, 124.1, 46.5, 26.7, 24.6, 22.6. HRMS (ESI) m/z: $[M+Na]^+$ Calcd for $C_{19}H_{18}N_2NaO_2S$ $[M+Na]^+$ 361.0981; Found 361.0985.

*N,2-Dimethyl-N,7-diphenylquinoline-3-sulfonamide (**5d**)*

Prepared from 3-azido-[1,1'-biphenyl]-4-carbaldehyde (**1d**) and *N*-methyl-2-oxo-*N*-phenylpropane-1-sulfonamide (**2a**) according to **GP1**. Eluent: hexane/ethyl acetate from 100:0 to 75:25. Yield: 192 mg (99%). Light yellow powder; mp 132-134°C. ^{1}H NMR (400 MHz, $CDCl_3$) δ 8.66 (s, 1H), 8.28 (d, *J* = 1.7 Hz, 1H), 7.91 (d, *J* = 8.5 Hz, 1H), 7.86 (dd, *J* = 8.5, 1.7 Hz, 1H), 7.78 – 7.73 (m, 2H), 7.55 – 7.48 (m, 2H), 7.47 – 7.41 (m, 1H), 7.35 – 7.26 (m, 3H), 7.24 – 7.20 (m, 2H), 3.33 (s, 3H), 2.72 (s, 3H). ^{13}C{^{1}H} NMR (101 MHz, $CDCl_3$) δ 156.4, 149.1, 145.4, 141.1, 140.1, 139.8, 130.4, 129.5, 129.3, 129.2, 128.6, 127.9, 127.6, 127.1, 127.1, 126.2, 124.4, 38.8, 24.8. HRMS (ESI) m/z: $[M+H]^+$ Calcd for $C_{23}H_{21}N_2O_2S$ $[M+H]^+$ 389.1318; Found 389.1320.

*7-Bromo-N,2-dimethyl-N-phenylquinoline-3-sulfonamide (**5e**)*

Prepared from 2-azido-4-bromobenzaldehyde (**1e**) and *N*-methyl-2-oxo-*N*-phenylpropane-1-sulfonamide (**2a**) according to **GP1**. Eluent: hexane/ethyl acetate from 100:0 to 85:15. Yield: 82 mg (42%). Yellow powder; mp 132-133°C. ^{1}H NMR (400 MHz, $CDCl_3$) δ 8.59 (s, 1H), 8.24 (d, *J* = 1.8 Hz, 1H), 7.70 (d, *J* = 8.6 Hz, 1H), 7.66 (dd, *J* = 8.6, 1.8 Hz, 1H), 7.33 – 7.27 (m, 3H), 7.21 – 7.14 (m, 2H), 3.31 (s, 3H), 2.69 (s, 3H). ^{13}C{^{1}H} NMR (101 MHz, $CDCl_3$) δ 157.3, 149.2, 140.9, 140.1, 131.3, 131.1, 131.1, 129.9, 129.5, 128.0, 127.2, 127.1, 124.0, 38.9, 24.8. HRMS (ESI) m/z: $[M+H]^+$ Calcd for $C_{17}H_{16}BrN_2O_2S$ $[M+H]^+$ 391.0110; Found 391.0108.

*N,2-Dimethyl-6-nitro-N-phenylquinoline-3-sulfonamide (**5f**)*

Prepared from 2-azido-5-nitrobenzaldehyde (**1f**) and *N*-methyl-2-oxo-*N*-phenylpropane-1-sulfonamide (**2a**) according to **GP1**. Eluent: hexane/ethyl acetate from 100:0 to 70:30. Yield: 10 mg (6%). Beige powder; mp 182°C. ^{1}H NMR (400 MHz, $CDCl_3$) δ 8.79 (d, *J* = 2.5 Hz, 1H), 8.78 (s, 1H), 8.57 (dd, *J* = 9.2, 2.5 Hz, 1H), 8.18 (d, *J* = 9.2 Hz, 1H), 7.35 – 7.28 (m, 3H), 7.22 – 7.17 (m, 2H), 3.36 (s, 3H), 2.77 (s, 3H). ^{13}C{^{1}H} NMR (126 MHz, $CDCl_3$) δ 160.2, 150.5, 146.0, 141.4, 140.6, 133.0, 130.7, 129.7, 128.3, 127.1, 125.8, 125.4, 124.4, 39.0, 25.2. HRMS (ESI) m/z: $[M+H]^+$ Calcd for $C_{17}H_{16}N_3O_4S$ $[M+H]^+$ 358.0856; Found 358.0853.

*6-Chloro-N,2-dimethyl-N-phenylquinoline-3-sulfonamide (**5g**)*

Prepared from 2-azido-5-chlorobenzaldehyde (**1g**) and *N*-methyl-2-oxo-*N*-phenylpropane-1-sulfonamide (**2a**) according to **GP1**. Eluent: hexane/ethyl acetate from 100:0 to 80:20. Yield: 73 mg (42%). White powder; mp 165-166°C. ^{1}H NMR (400 MHz, $CDCl_3$) δ 8.54 (s, 1H), 7.98 (d, *J* = 9.0 Hz, 1H), 7.81 (d, *J* = 2.4 Hz, 1H), 7.74 (dd, *J* = 9.0, 2.4 Hz, 1H), 7.34 – 7.27 (m, 3H), 7.22 – 7.10 (m, 2H), 3.31 (s, 3H), 2.69 (s, 3H). ^{13}C{^{1}H} NMR (101 MHz, $CDCl_3$) δ 156.3, 147.1, 140.9, 139.2, 133.4, 133.2, 131.7, 130.3, 129.5, 128.0, 127.3, 127.1, 126.0, 38.9, 24.7. HRMS (ESI) m/z: $[M+H]^+$ Calcd for $C_{17}H_{16}ClN_2O_2S$ $[M+H]^+$ 347.0616; Found 347.0624.

*N,2-Dimethyl-5-oxo-N-phenyl-5H-chromeno[4,3-b]pyridine-3-sulfonamide (**5h**)*

Prepared from 4-azido-2-oxo-2*H*-chromene-3-carbaldehyde (**1h**) and *N*-methyl-2-oxo-*N*-phenylpropane-1-sulfonamide (**2a**) according to **GP1**. Eluent: hexane/ethyl acetate from 100:0 to 80:20. Yield: 45 mg (47%). White powder; mp 162-163°C. ^{1}H NMR (400 MHz, $CDCl_3$) δ 8.94 (s, 1H), 8.57 (d, *J* = 7.9 Hz, 1H), 7.64 (t, *J* = 7.9 Hz, 1H), 7.44 – 7.37 (m, 2H), 7.36 – 7.29 (m, 3H), 7.21 (d, *J* = 7.4 Hz, 2H), 3.34 (s, 3H), 2.65 (s, 3H). ^{13}C{^{1}H} NMR (101 MHz, $CDCl_3$) δ 164.7, 160.0, 153.6, 153.4, 140.7, 140.7, 133.7, 133.4, 129.6, 128.2, 127.0, 125.7, 125.3, 118.4, 117.5, 114.9, 39.0, 25.1. HRMS (ESI) m/z: $[M+Na]^+$ Calcd for $C_{20}H_{16}N_2NaO_4S$ $[M+Na]^+$ 403.0723; Found 403.0727.

*2-Methyl-N-phenylquinoline-3-sulfonamide (**5i**)*

Prepared from 2-azidobenzaldehyde (**1a**) and 2-oxo-*N*-phenylpropane-1-sulfonamide (**2i**) according to **GP2**. Eluent: hexane/ethyl acetate from 100:0 to 65:35. Yield: 94 mg (79%). White powder; mp 186-187°C. ^{1}H NMR (400 MHz, DMSO-d_6) δ 10.7 (s, 1H), 9.0 (s, 1H), 8.2 (dd, *J* = 8.4, 1.3 Hz, 1H), 8.0 (d, *J* = 8.2 Hz, 1H), 7.9 (ddd, *J* = 8.4, 6.8, 1.5 Hz, 1H), 7.6 (ddd, *J* = 8.2, 6.8, 1.3 Hz, 1H), 7.2 – 7.2 (m, 2H), 7.2 – 7.1 (m, 2H), 7.0 – 6.9 (m, 1H), 2.9 (s, 3H). ^{13}C{^{1}H} NMR (101 MHz, DMSO-d_6) δ 154.4, 147.9, 139.5, 137.0, 132.6, 131.7, 129.3, 129.2, 127.9, 127.3, 124.9, 124.0, 119.3, 23.8. HRMS (ESI) m/z: $[M+Na]^+$ Calcd for $C_{16}H_{14}N_2NaO_2S$ $[M+Na]^+$ 321.0668; Found 321.0665.

*N-Benzyl-2-methylquinoline-3-sulfonamide (**5j**)*

Prepared from 2-azidobenzaldehyde (**1a**) and *N*-benzyl-2-oxopropane-1-sulfonamide (**2j**) according to **GP2**. Eluent: hexane/ethyl acetate from 100:0 to 65:35. Yield: 105 mg (84%). White powder; mp 129°C. ^{1}H NMR (400 MHz, DMSO-d_6) δ 8.76 (s, 1H), 8.61 (t, *J* = 6.1 Hz, 1H), 8.10 (d, *J* = 8.2 Hz, 1H), 7.98 (d, *J* = 8.4 Hz, 1H), 7.87 (ddd, *J* = 8.2, 6.9, 1.3 Hz, 1H), 7.69 – 7.61 (m, 1H), 7.16 (dd, *J* = 13.1, 5.8 Hz, 4H), 7.08 (t, *J* = 7.0 Hz, 1H), 4.12 (d, *J* = 6.1 Hz, 2H), 2.90 (s, 3H). ^{13}C{^{1}H} NMR (101 MHz, DMSO-d_6) δ 154.6, 147.6, 137.8, 137.4, 133.2, 132.1, 129.1, 128.1, 127.8, 127.5, 127.0, 125.1, 45.9, 23.8. HRMS (ESI) m/z: $[M+Na]^+$ Calcd for $C_{17}H_{16}N_2NaO_2S$ $[M+Na]^+$ 335.0825; Found 335.0830.

*2-Methyl-N-(o-tolyl)quinoline-3-sulfonamide (**5k**)*

Prepared from 2-azidobenzaldehyde (**1a**) and 2-oxo-*N*-(*o*-tolyl)propane-1-sulfonamide (**2k**) according to **GP2**. Eluent: hexane/ethyl acetate from 100:0 to 65:35. Yield: 106 mg (85%). Beige powder; mp 155-156°C. ^{1}H NMR (400 MHz, $CDCl_3$) δ 8.72 (s, 1H), 8.06 (d, *J* = 8.6 Hz, 1H), 7.86 – 7.79 (m, 2H), 7.57 (t, *J* = 7.5 Hz, 1H), 7.16 – 6.99 (m, 4H), 6.81 (s, 1H), 3.03 (s, 3H), 2.21 (s, 3H).

$^{13}C\{^1H\}$ NMR (101 MHz, $CDCl_3$) δ 155.0, 148.8, 139.7, 134.0, 132.7, 132.6, 131.9, 131.3, 129.0, 128.7, 127.5, 127.1, 126.7, 125.5, 124.1, 24.5, 18.0. HRMS (ESI) m/z: $[M+H]^+$ Calcd for $C_{17}H_{17}N_2O_2S$ $[M+H]^+$ 313.1005; Found 313.1003.

*2-Ethoxy-N-phenylquinoline-3-sulfonamide (**5l**)*

Prepared from 2-azidobenzaldehyde (**1a**) and ethyl 2-((phenylamino)sulfinyl)acetate (**2l**) according to **GP2**. Eluent: hexane/ethyl acetate from 100:0 to 80:20. Yield: 83 mg (63%). White powder; mp 203-204°C. ^1H NMR (400 MHz, DMSO-d_6) δ 10.26 (s, 1H), 8.89 (s, 1H), 8.09 (d, J = 8.1 Hz, 1H), 7.84 – 7.69 (m, 2H), 7.49 (ddd, J = 8.1, 6.6, 1.5 Hz, 1H), 7.27 – 7.07 (m, 4H), 6.95 (t, J = 7.0 Hz, 1H), 4.59 (q, J = 7.0 Hz, 2H), 1.38 (t, J = 7.0 Hz, 3H). $^{13}C\{^1H\}$ NMR (101 MHz, DMSO-d_6) δ 155.9, 147.2, 142.6, 137.3, 132.8, 129.4, 129.0, 126.3, 125.2, 123.9, 123.2, 122.9, 119.6, 62.4, 14.2. HRMS (ESI) m/z: $[M+Na]^+$ Calcd for $C_{17}H_{16}N_2NaO_3S$ $[M+Na]^+$ 351.0774; Found 351.0785.

*N-(4-Methoxyphenyl)-2-methylquinoline-3-sulfonamide (**5m**)*

Prepared from 2-azidobenzaldehyde (**1a**) and *N*-(4-methoxyphenyl)-2-oxopropane-1-sulfonamide (**2m**) according to **GP2**. Eluent: hexane/ethyl acetate from 100:0 to 65:35. Yield: 113 mg (86%). Light orange oil. ^1H NMR (400 MHz, $CDCl_3$) δ 8.67 (s, 1H), 8.05 (d, J = 8.8 Hz, 1H), 7.84 – 7.79 (m, 2H), 7.56 (ddd, J = 8.1, 6.9, 1.2 Hz, 1H), 7.01 – 6.96 (m, 2H), 6.87 (s, 1H), 6.74 – 6.68 (m, 2H), 3.69 (s, 3H), 3.04 (s, 3H). $^{13}C\{^1H\}$ NMR (101 MHz, $CDCl_3$) δ 158.4, 155.0, 148.8, 140.1, 132.7, 131.9, 129.0, 128.6, 128.0, 127.5, 125.8, 125.5, 114.8, 55.5, 24.5. HRMS (ESI) m/z: $[M+Na]^+$ Calcd for $C_{17}H_{16}N_2NaO_3S$ $[M+Na]^+$ 351.0774; Found 351.0776.

*2-Methyl-N-(4-(trifluoromethyl)phenyl)quinoline-3-sulfonamide (**5n**)*

Prepared from 2-azidobenzaldehyde (**1a**) and 2-oxo-*N*-(4-(trifluoromethyl)phenyl)propane-1-sulfonamide (**2n**) according to **GP2**. Eluent: hexane/ethyl acetate from 100:0 to 75:25. Yield: 93 mg (63%). Yellow oil. ^1H NMR (400 MHz, $CDCl_3$) δ 8.86 (s, 1H), 8.06 (dd, J = 8.4, 1.2 Hz, 2H), 7.90 (dd, J = 8.2, 1.5 Hz, 1H), 7.84 (td, J = 8.4, 6.9, 1.5 Hz, 2H), 7.61 (ddd, J = 8.2, 6.9, 1.2 Hz, 2H), 7.46 (d, J = 8.4 Hz, 2H), 7.20 (d, J = 8.4 Hz, 2H). $^{13}C\{^1H\}$ NMR (101 MHz, $CDCl_3$) δ 154.6, 148.9, 140.2, 139.4, 133.2, 131.5, 129.0, 128.7, 127.8, 127.0 (q, C-F, $^3J_{C\text{-}F}$ = 3.9 Hz), 125.4, 123.9 (q, C-F, $^1J_{C\text{-}F}$ = 271.6 Hz), 119.4, 24.3. $^{19}F\{^1H\}$ NMR (376 MHz, $CDCl_3$) δ -62.4. HRMS (ESI) m/z: $[M+H]^+$ Calcd for $C_{17}H_{14}F_3N_2O_2S$ $[M+H]^+$ 367.0723; Found 367.0729.

*2-Methyl-3-tosylquinoline (**5o**)*

Prepared from 2-azidobenzaldehyde (**1a**) and 1-tosylpropan-2-one (**2o**) according to **GP1**. Eluent: hexane/ethyl acetate from 100:0 to 75:25. Yield: 109 mg (73%). Light yellow powder; mp 151-152°C. The spectral characteristics matched the previously reported data [9]. ^1H NMR (400 MHz, $CDCl_3$) δ 9.03 (s, 1H), 8.03 (dd, J = 8.5, 1.0 Hz, 1H), 7.97 (dd, J = 8.2, 1.3 Hz, 1H), 7.85 – 7.82 (m, 1H), 7.80 (d, J = 8.2 Hz, 2H), 7.61 (ddd, J = 8.2, 6.9, 1.0 Hz, 1H), 7.31 (d, J = 8.2 Hz, 2H), 2.79 (s, 3H), 2.41 (s, 3H). $^{13}C\{^1H\}$ NMR (101 MHz, $CDCl_3$) δ 155.4, 149.2, 144.8, 139.2, 137.4, 134.0, 132.8, 130.0, 129.1, 128.8, 128.2, 127.5, 125.7, 24.4, 21.7. HRMS (ESI) m/z: $[M+Na]^+$ Calcd for $C_{17}H_{15}NNaO_2S$ $[M+Na]^+$ 320.0716; Found 320.0711.

*2-Phenyl-3-tosylquinoline (**5p**)*

Prepared from 2-azidobenzaldehyde (**1a**) and 1-phenyl-2-tosylethan-1-one (**2p**) according to **GP1**. Yield: 119 mg (66%). Beige powder; mp 248°C. The spectral characteristics matched the previously reported data [10]. ^{1}H NMR (400 MHz, $CDCl_3$) δ 9.29 (s, 1H), 8.16 (dd, *J* = 8.5, 1.0 Hz, 2H), 8.08 (dd, *J* = 8.2, 1.5 Hz, 2H), 7.89 (ddd, *J* = 8.5, 6.9, 1.5 Hz, 1H), 7.71 (td, *J* = 8.2, 6.9, 1.0 Hz, 2H), 7.40 (t, *J* = 7.4 Hz, 1H), 7.32 – 7.25 (m, 2H), 7.20 (d, *J* = 7.4 Hz, 2H), 7.13 (d, *J* = 8.1 Hz, 2H), 6.99 (d, *J* = 8.1 Hz, 2H), 2.33 (s, 3H). $^{13}C\{^{1}H\}$ NMR (101 MHz, $CDCl_3$) δ 157.3, 148.8, 144.1, 139.1, 138.4, 137.0, 135.1, 132.9, 129.8, 129.5, 129.2, 129.1, 128.7, 128.2, 128.1, 127.6, 125.9, 21.7. HRMS (ESI) m/z: $[M+Na]^+$ Calcd for $C_{22}H_{17}NNaO_2S$ $[M+Na]^+$ 382.0872; Found 382.0870.

*2-(Allyloxy)-3-tosylquinoline (**5q**)*

Prepared from 2-azidobenzaldehyde (**1a**) and allyl 2-tosylacetate (**2q**) according to **GP1**. Eluent: hexane/ethyl acetate from 100:0 to 90:10. Yield: 87 mg (51%). Light yellow powder; mp 150-151°C. ^{1}H NMR (400 MHz, $CDCl_3$) δ 8.94 (s, 1H), 7.92 (d, *J* = 8.3 Hz, 2H), 7.89 (dd, *J* = 8.2, 1.5 Hz, 1H), 7.80 (d, *J* = 8.4 Hz, 1H), 7.73 (ddd, *J* = 8.5, 6.8, 1.5 Hz, 1H), 7.47 (ddd, *J* = 8.2, 6.8, 1.3 Hz, 1H), 7.28 (d, *J* = 8.3 Hz, 2H), 6.00 (ddt, *J* = 17.2, 10.4, 5.7 Hz, 1H), 5.33 (ddd, *J* = 17.2, 3.0, 1.6 Hz, 1H), 5.24 (ddd, *J* = 10.4, 3.0, 1.4 Hz, 1H), 4.97 (ddd, *J* = 5.7, 1.6, 1.4 Hz, 2H), 2.41 (s, 3H). $^{13}C\{^{1}H\}$ NMR (101 MHz, $CDCl_3$) δ 156.2, 148.5, 144.5, 141.4, 137.4, 132.8, 132.5, 129.4, 129.3, 129.2, 127.2, 125.7, 125.5, 124.0, 118.5, 67.5, 21.7. HRMS (ESI) m/z: $[M+Na]^+$ Calcd for $C_{19}H_{17}NNaO_3S$ $[M+Na]^+$ 362.0821; Found 362.0823.

V. NMR spectra

Copies of ^{1}H (400.13 MHz, $CDCl_3$) and $^{13}C\{^{1}H\}$ (100.61 MHz, $CDCl_3$) of **5a**

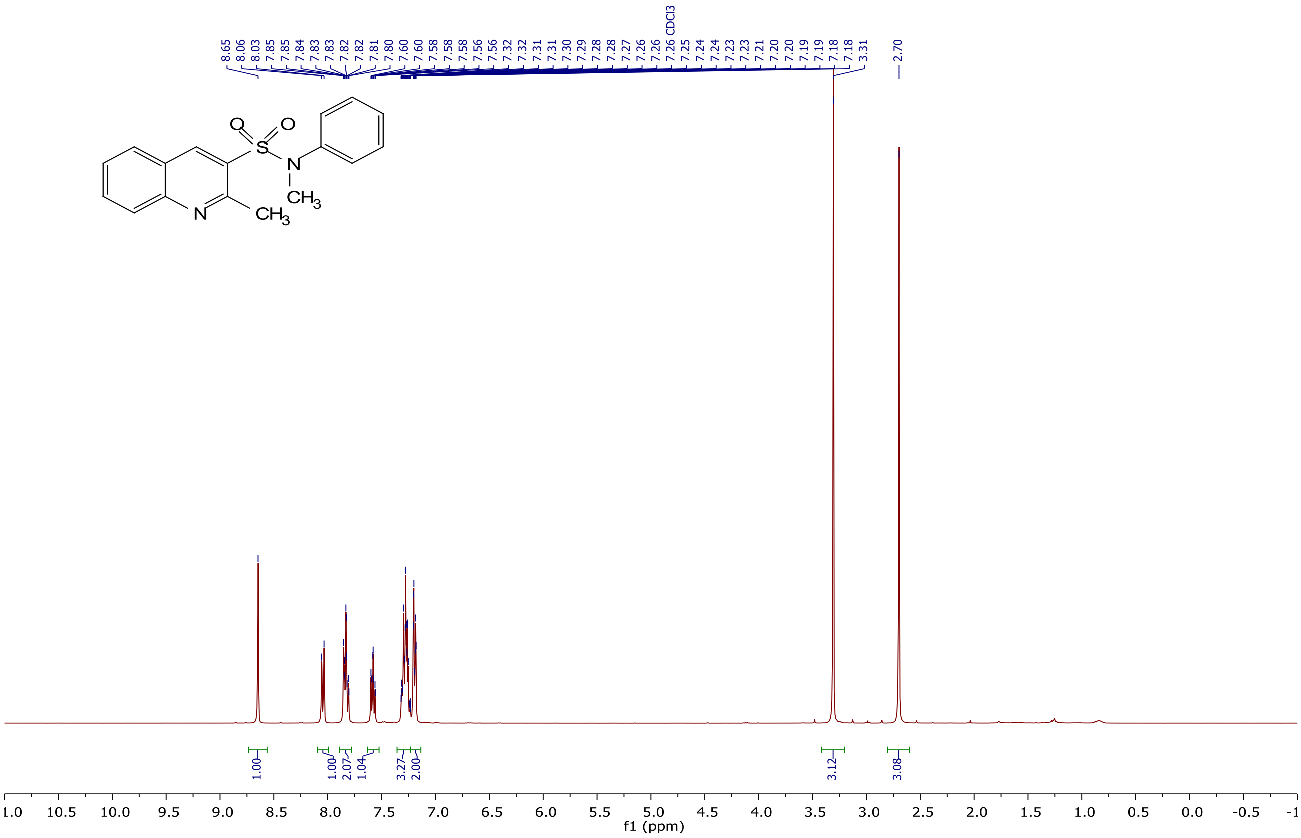

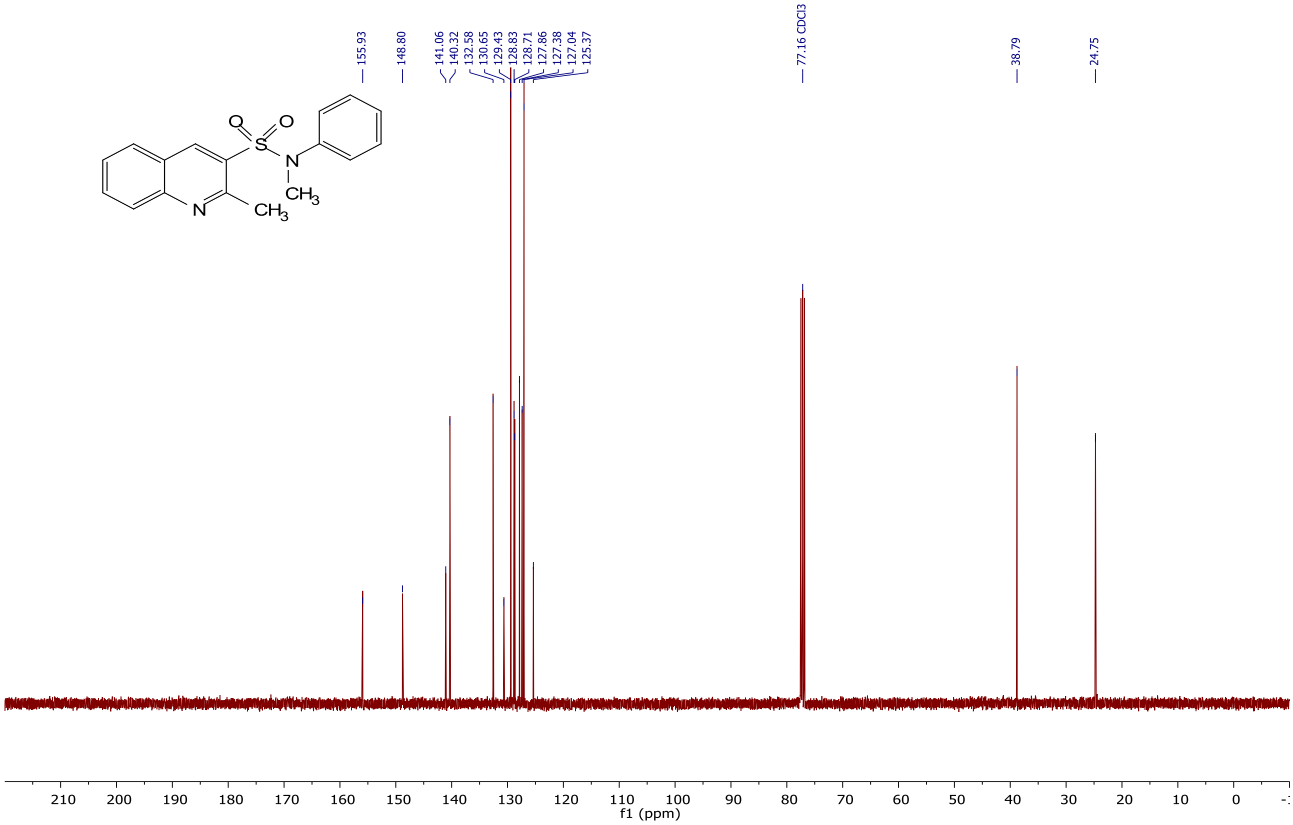

Copies of ^{1}H (400.13 MHz, $CDCl_3$) and $^{13}C\{^{1}H\}$ (100.61 MHz, $CDCl_3$) of **5b**

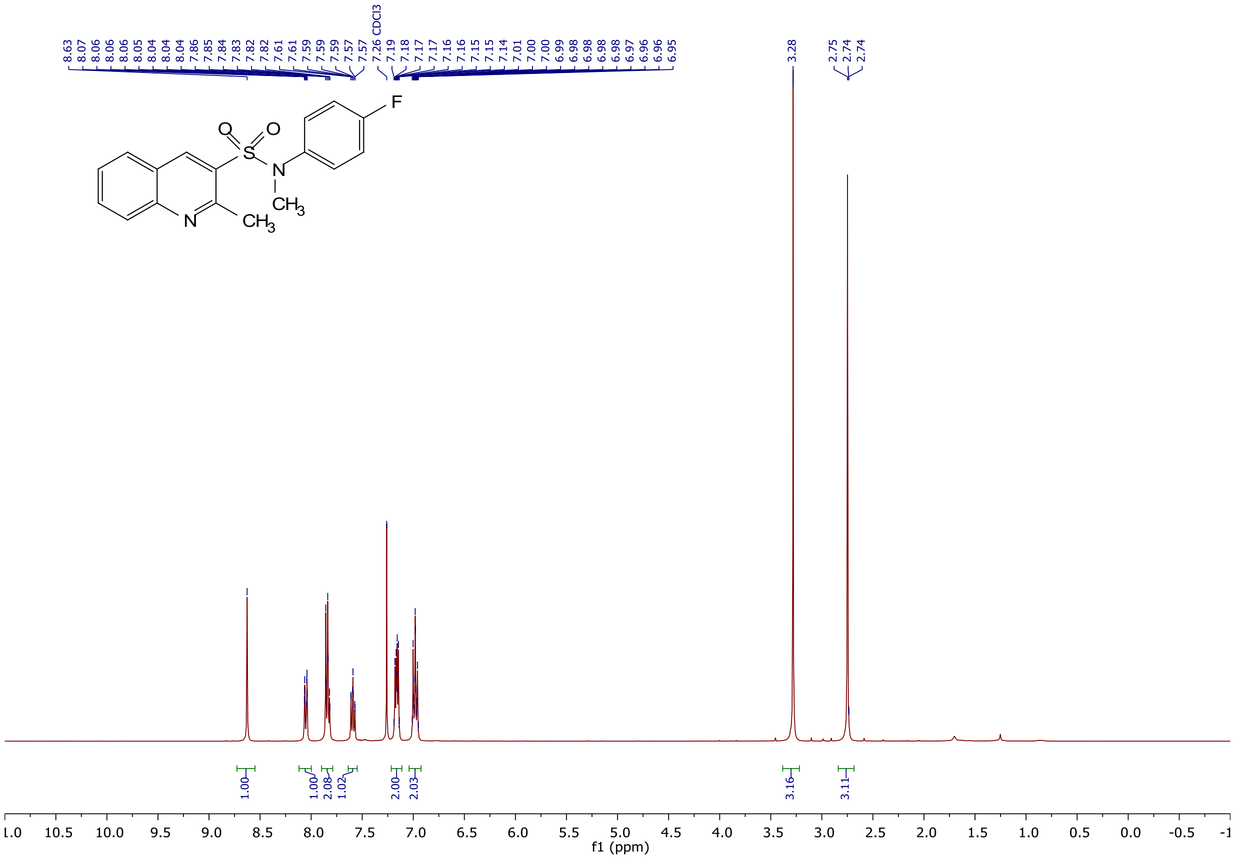

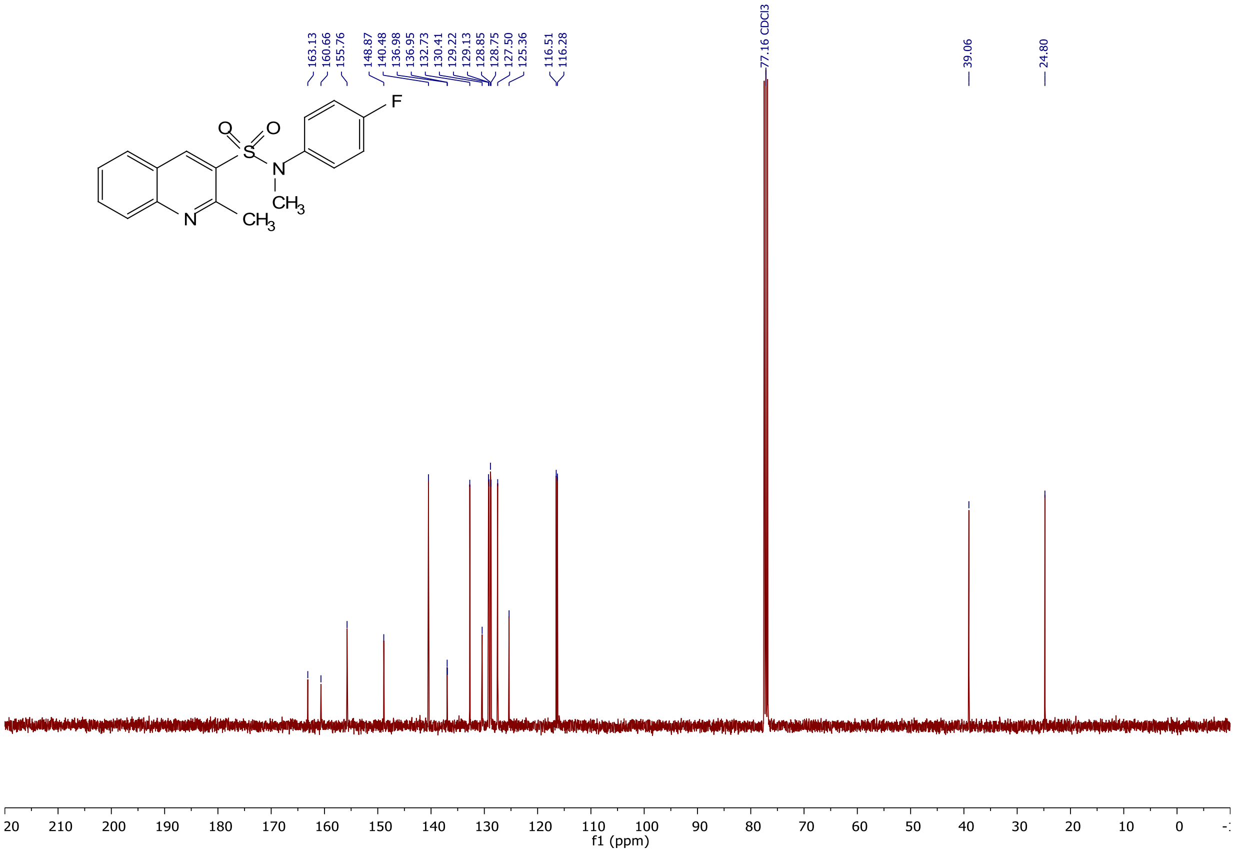

Copy of $^{19}F\{^{1}H\}$ (376.50 MHz, $CDCl_3$) spectrum of **5b**

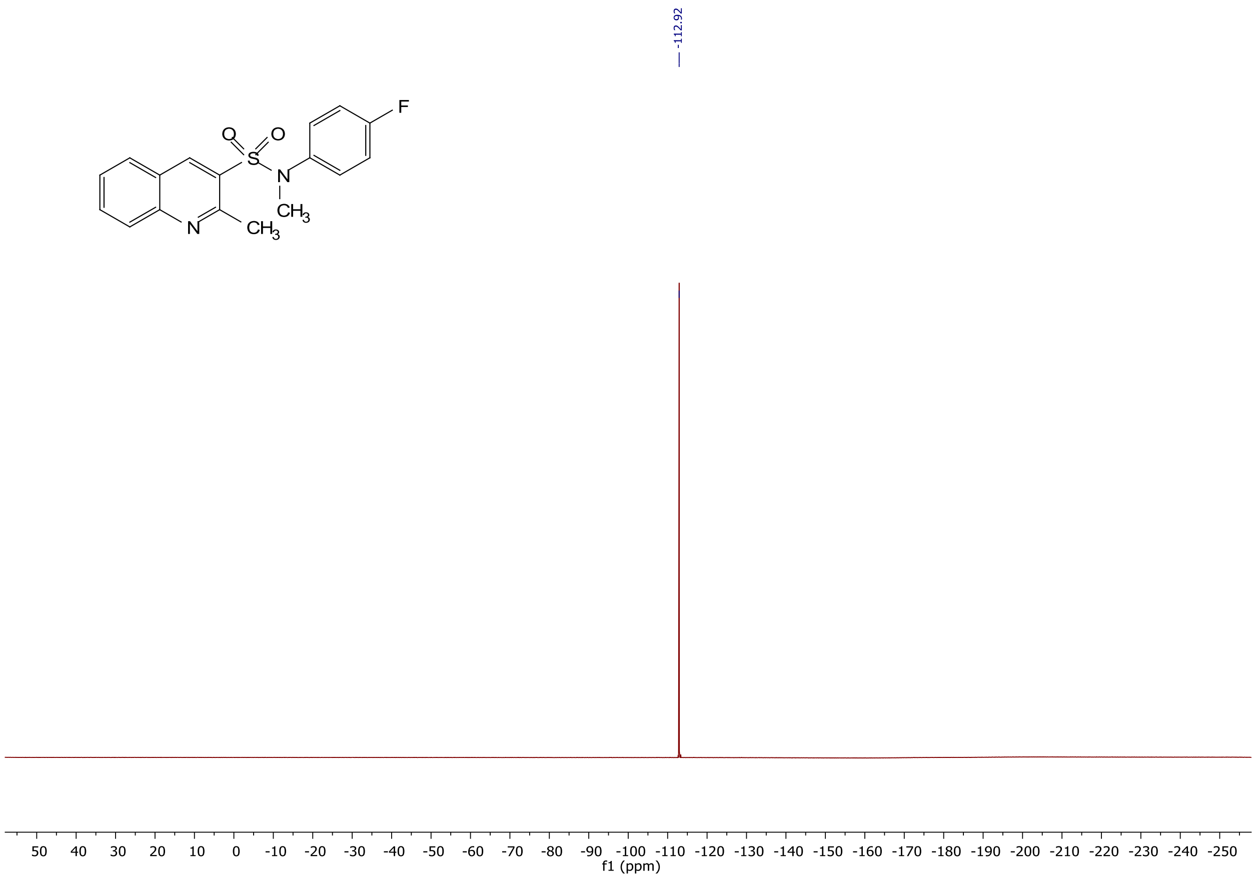

Copies of ^{1}H (400.13 MHz, $CDCl_3$) and $^{13}C\{^{1}H\}$ (100.61 MHz, $CDCl_3$) of **5c**

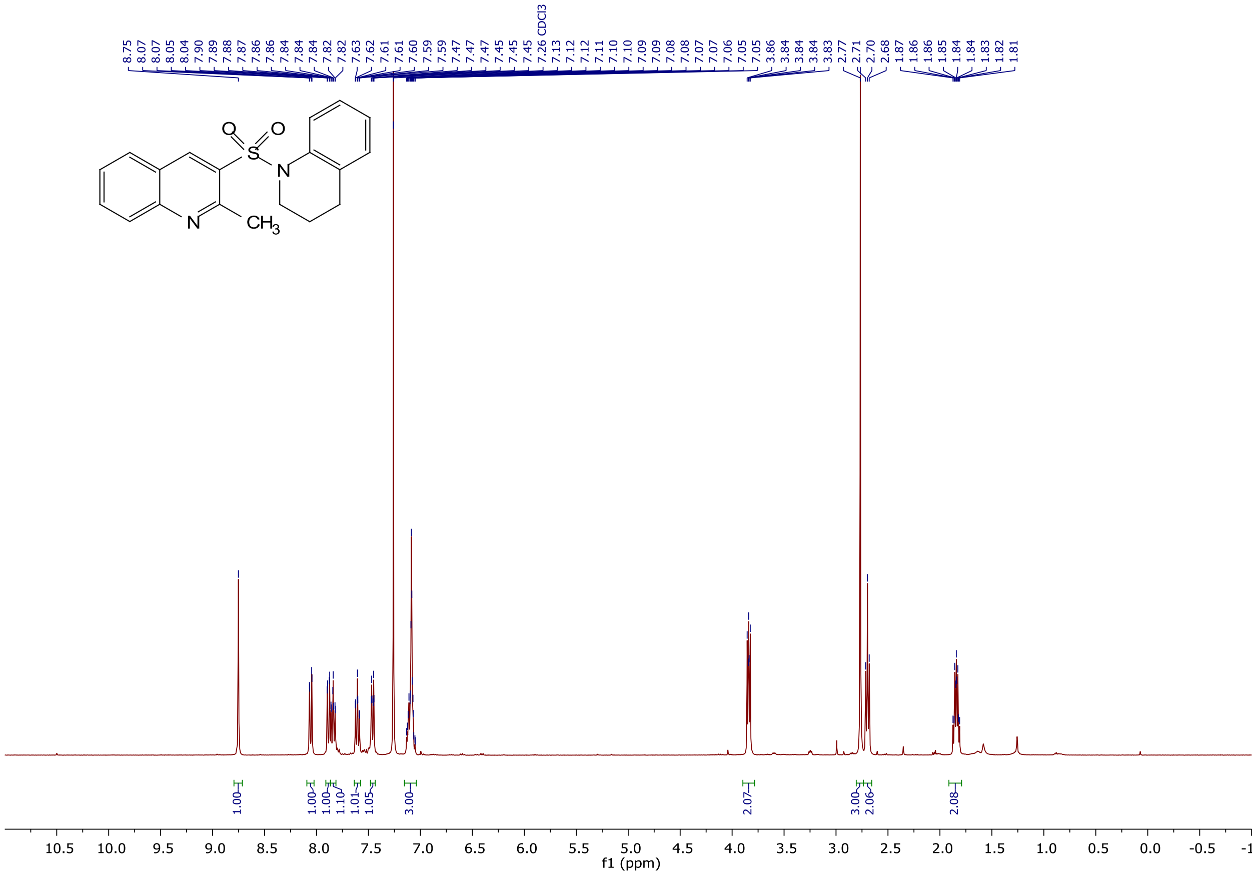

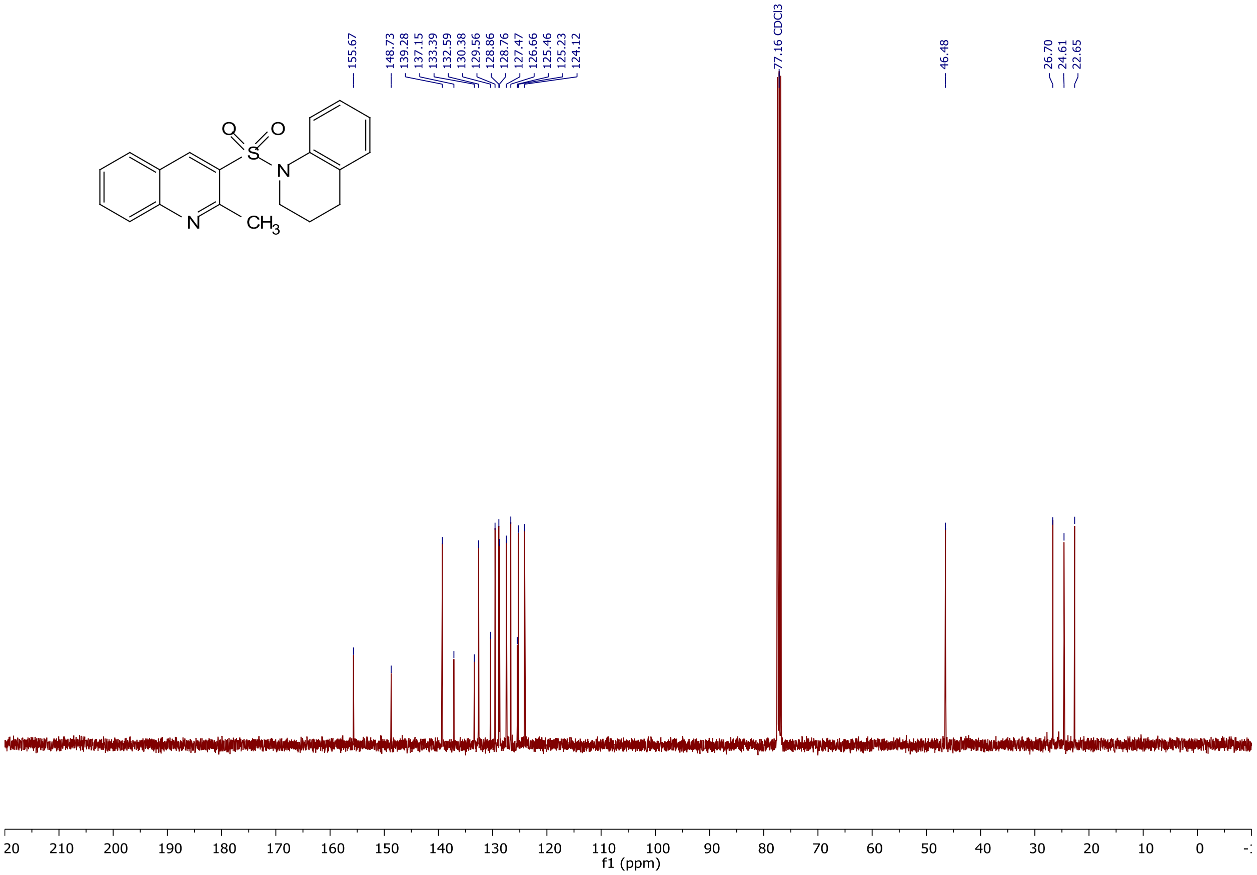

Copies of ^{1}H (400.13 MHz, $CDCl_3$) and $^{13}C\{^{1}H\}$ (100.61 MHz, $CDCl_3$) of **5d**

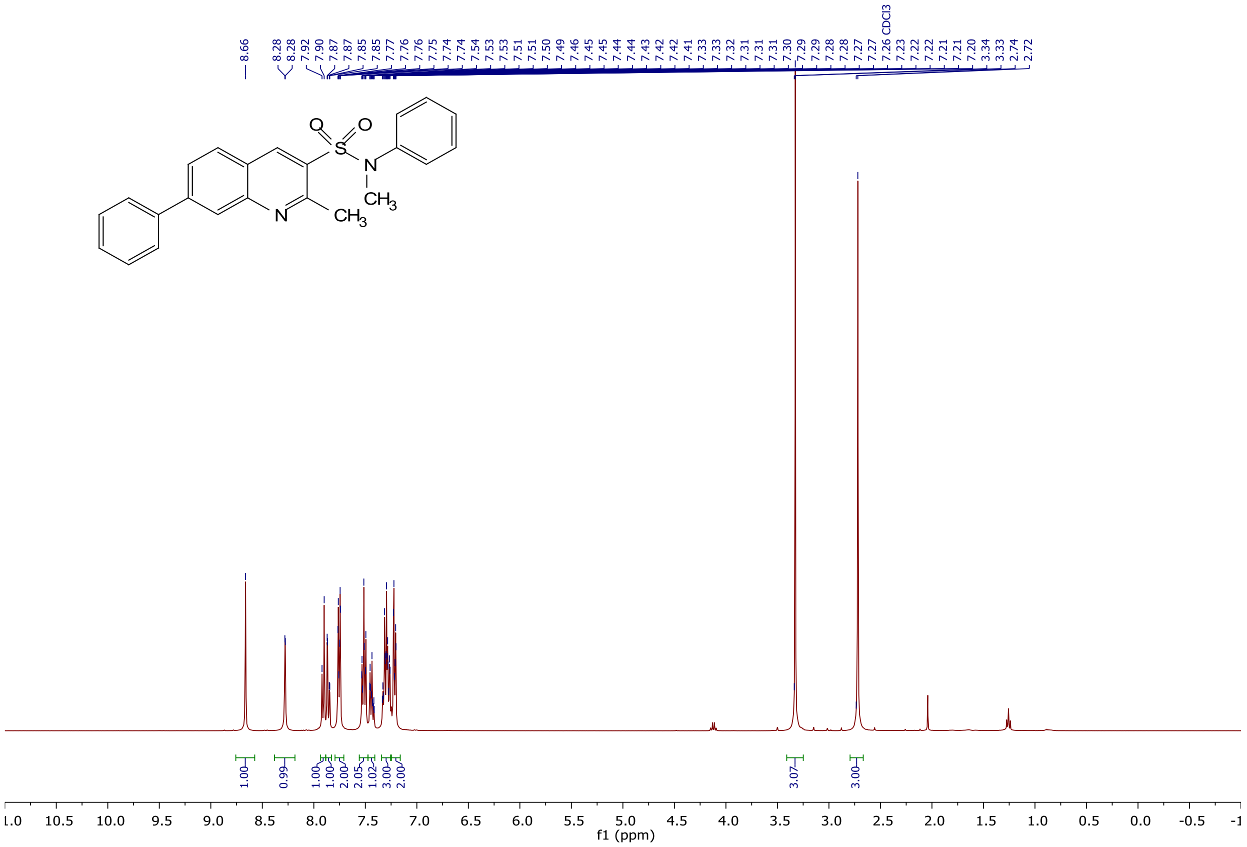

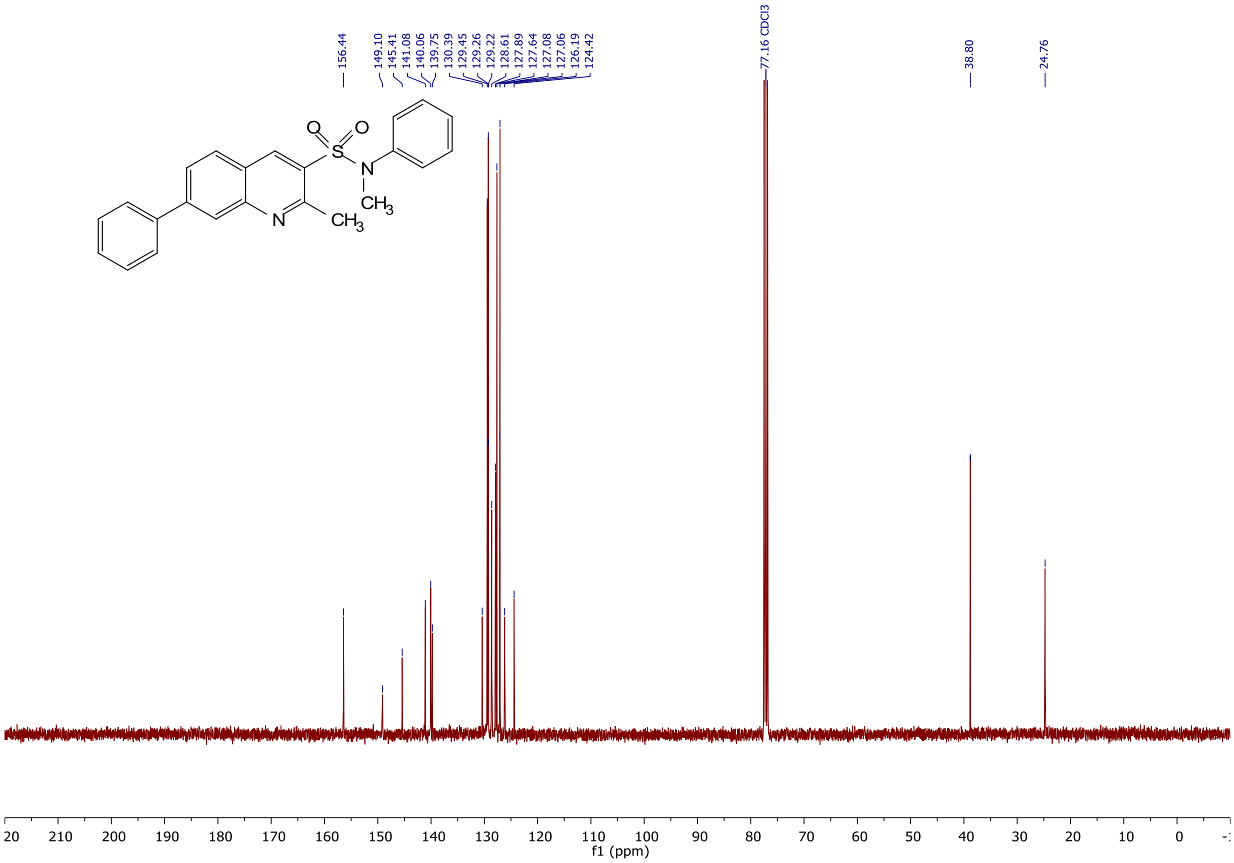

Copies of ^{1}H (400.13 MHz, $CDCl_3$) and $^{13}C\{^{1}H\}$ (100.61 MHz, $CDCl_3$) of **5e**

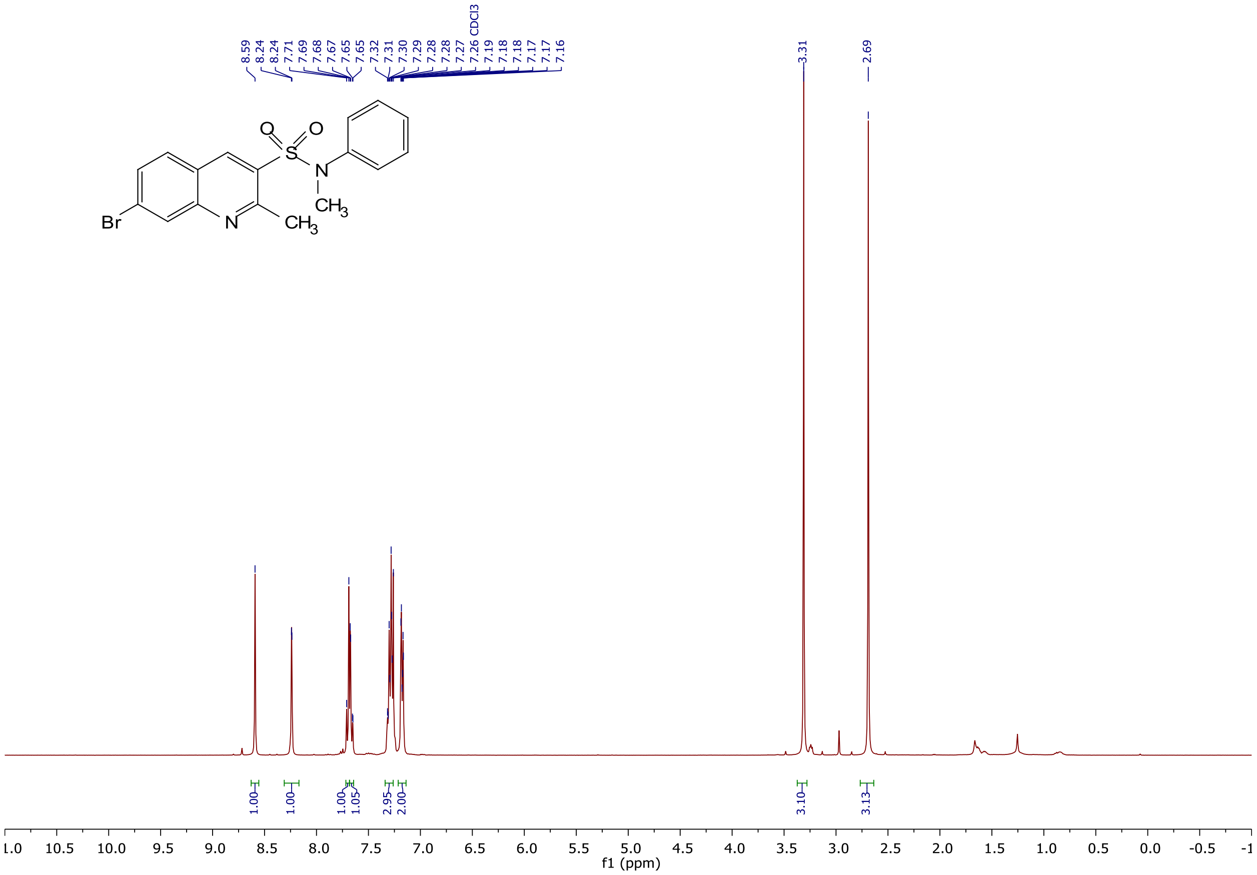

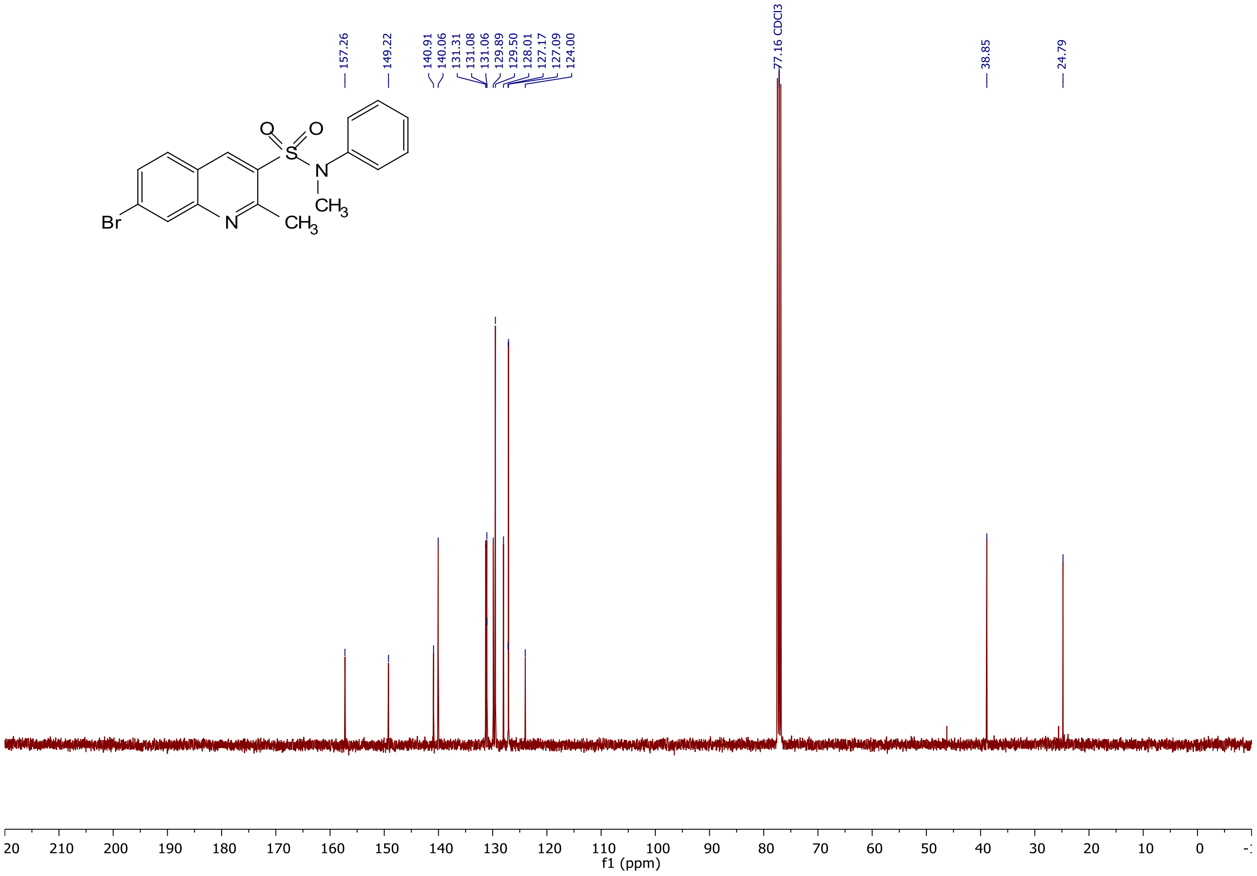

Copies of ^{1}H (400.13 MHz, $CDCl_3$) and $^{13}C\{^{1}H\}$ (125.75 MHz, $CDCl_3$) of **5f**

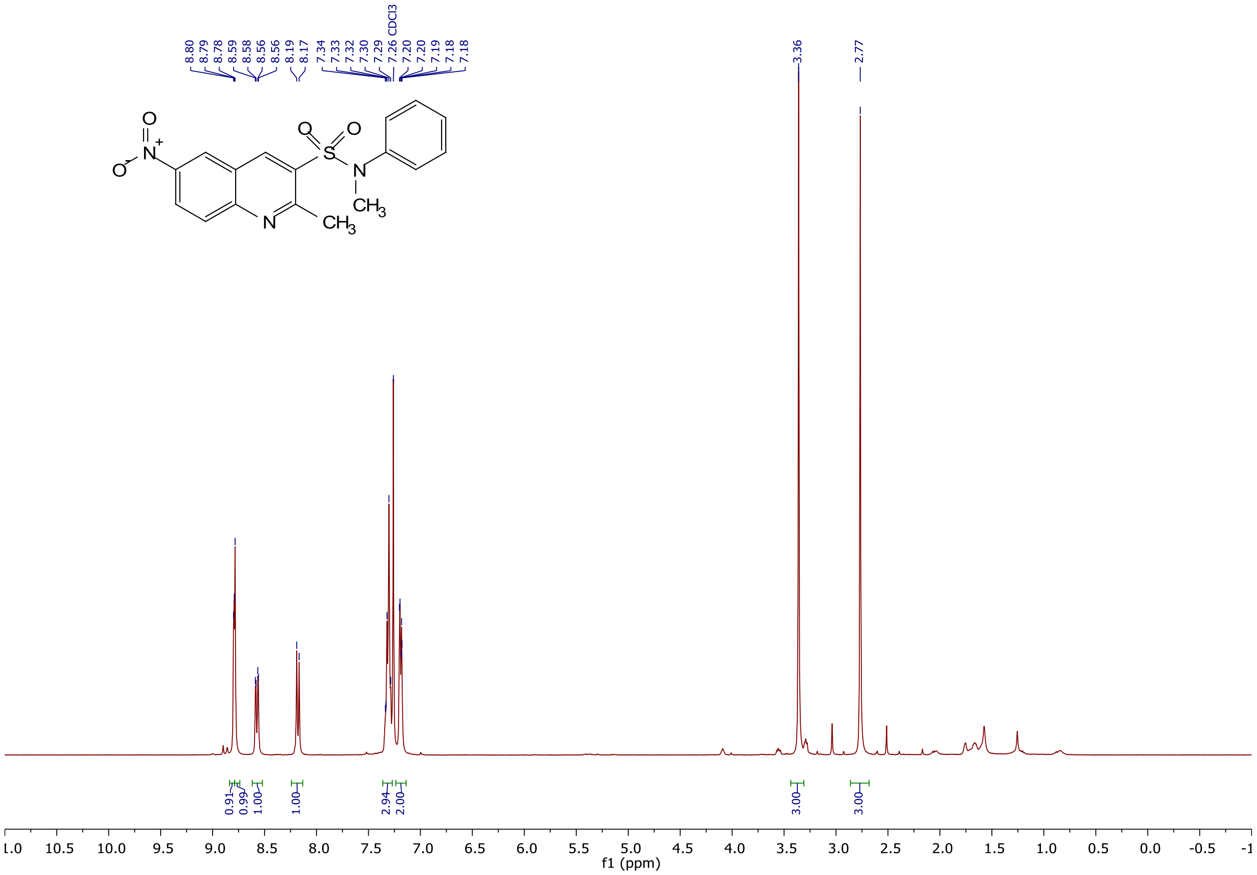

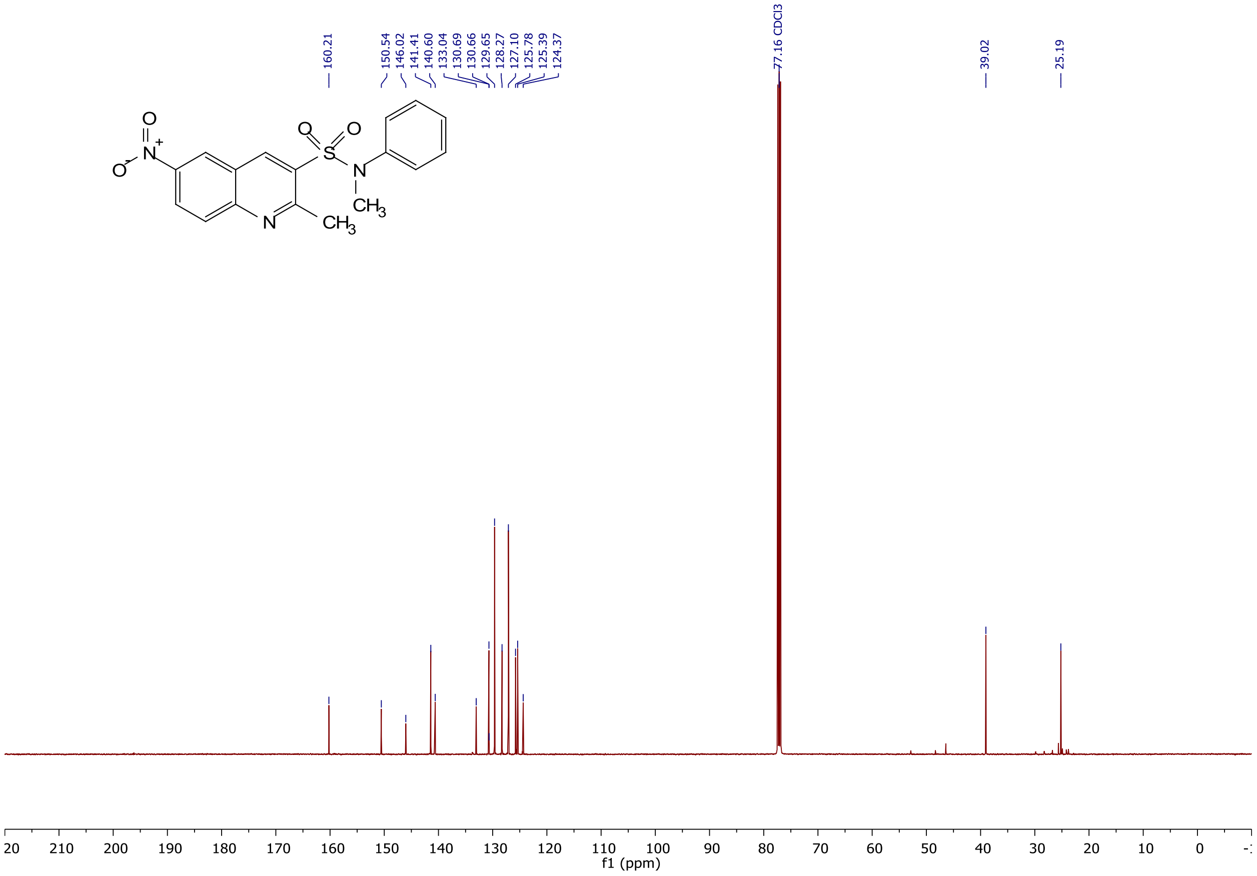

Copies of ^{1}H (400.13 MHz, $CDCl_3$) and $^{13}C\{^{1}H\}$ (100.61 MHz, $CDCl_3$) of **5g**

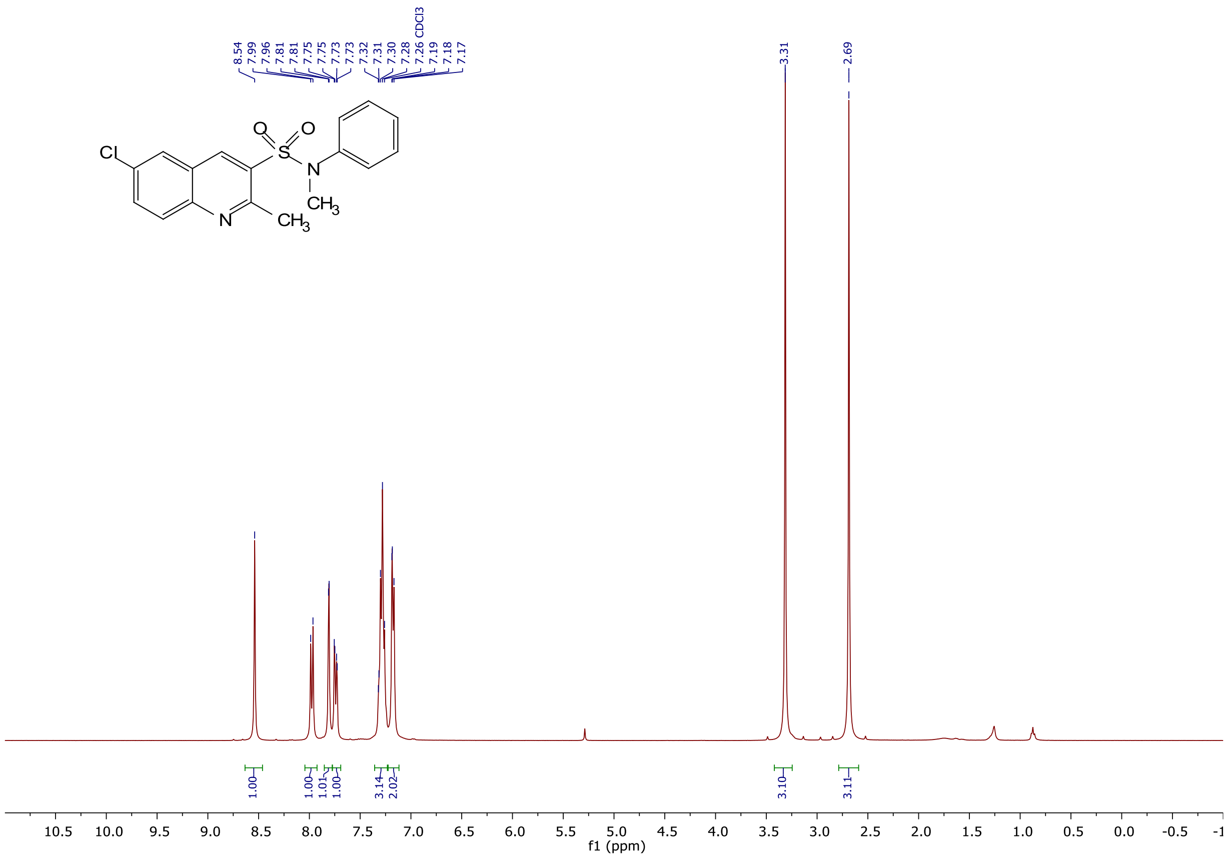

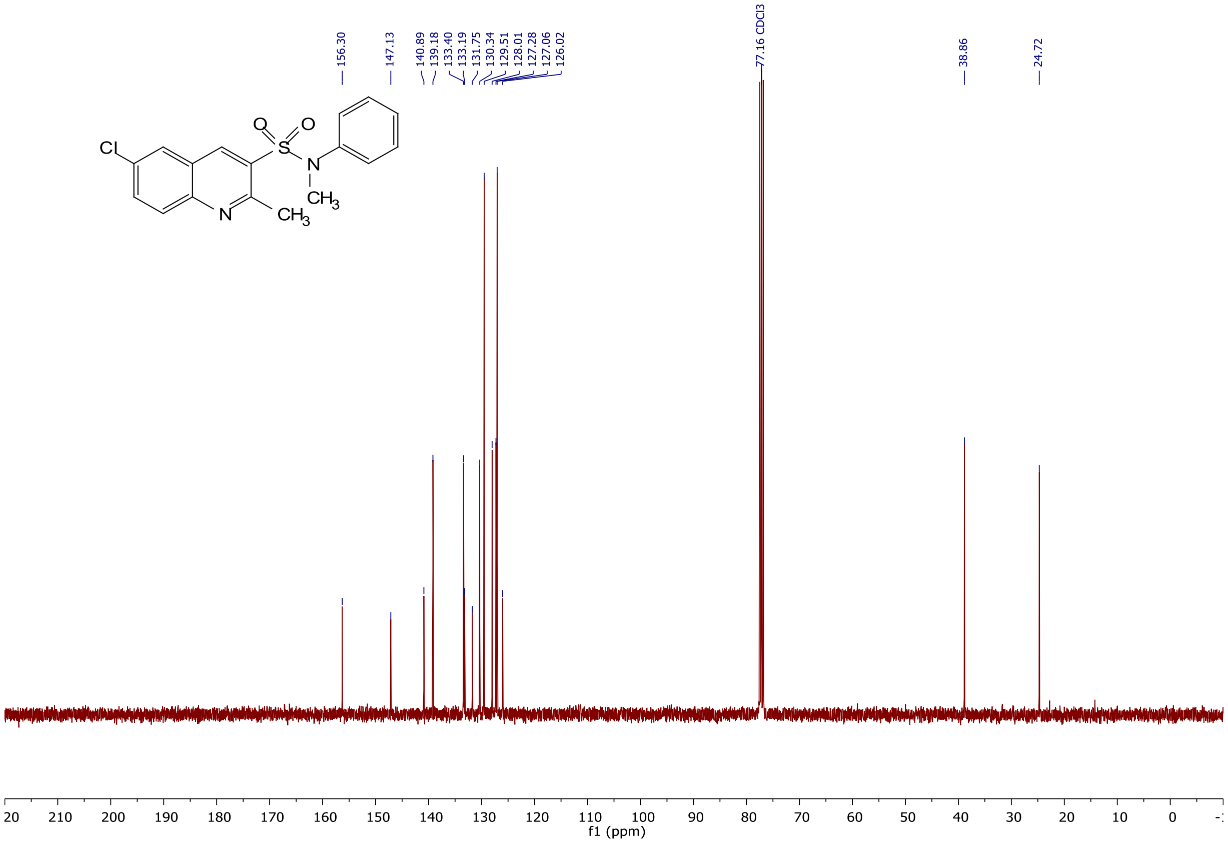

Copies of ^{1}H (400.13 MHz, $CDCl_3$) and $^{13}C\{^{1}H\}$ (100.61 MHz, $CDCl_3$) of **5h**

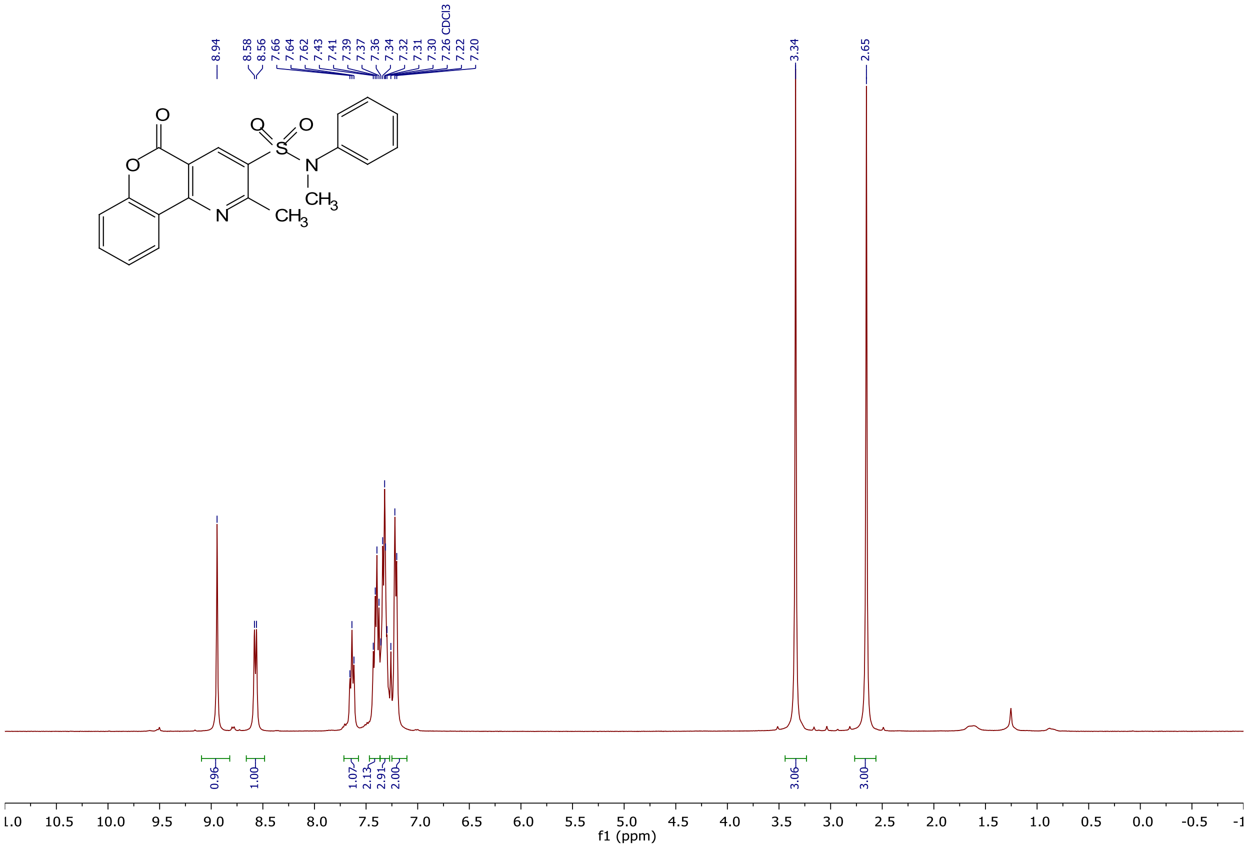

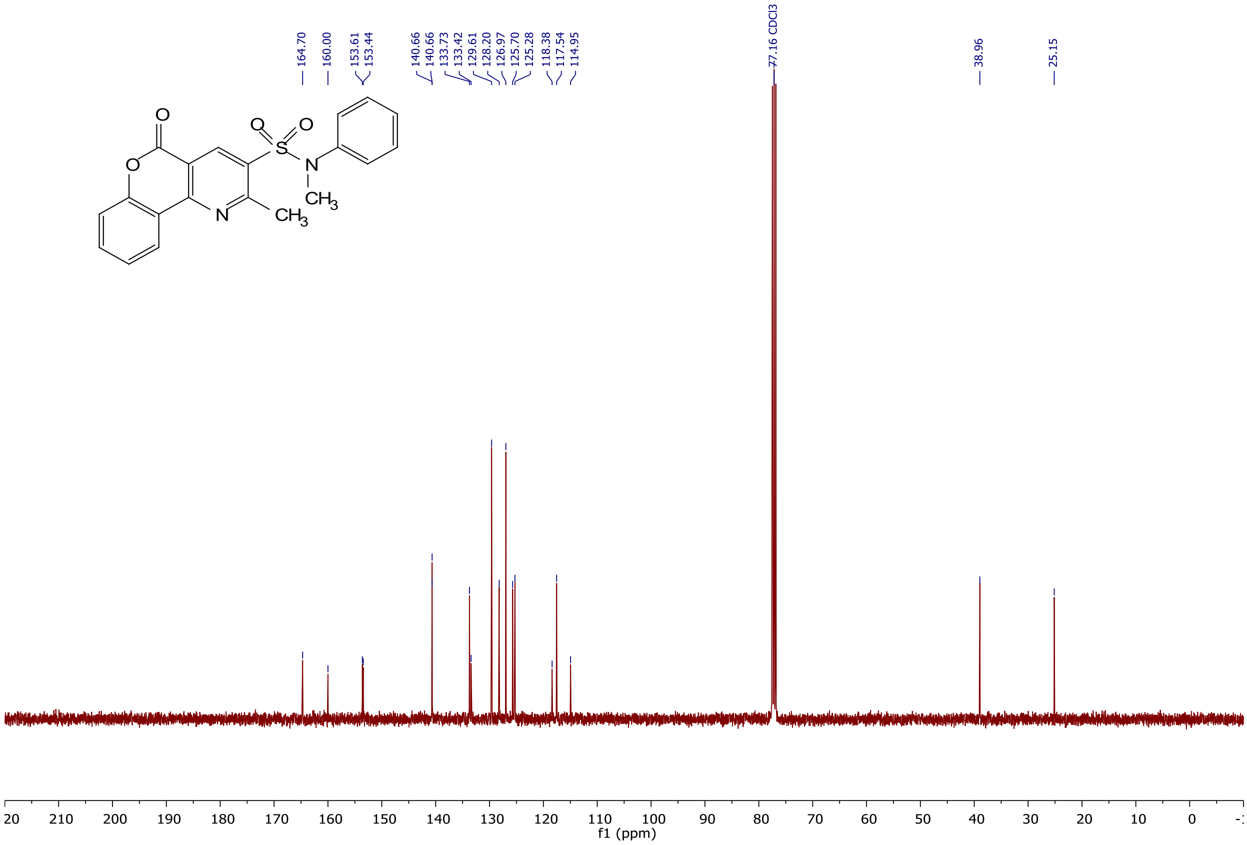

Copies of ^{1}H (400.13 MHz, DMSO-d_6) and ^{13}C{^{1}H} (100.61 MHz, DMSO-d_6) of **5i**

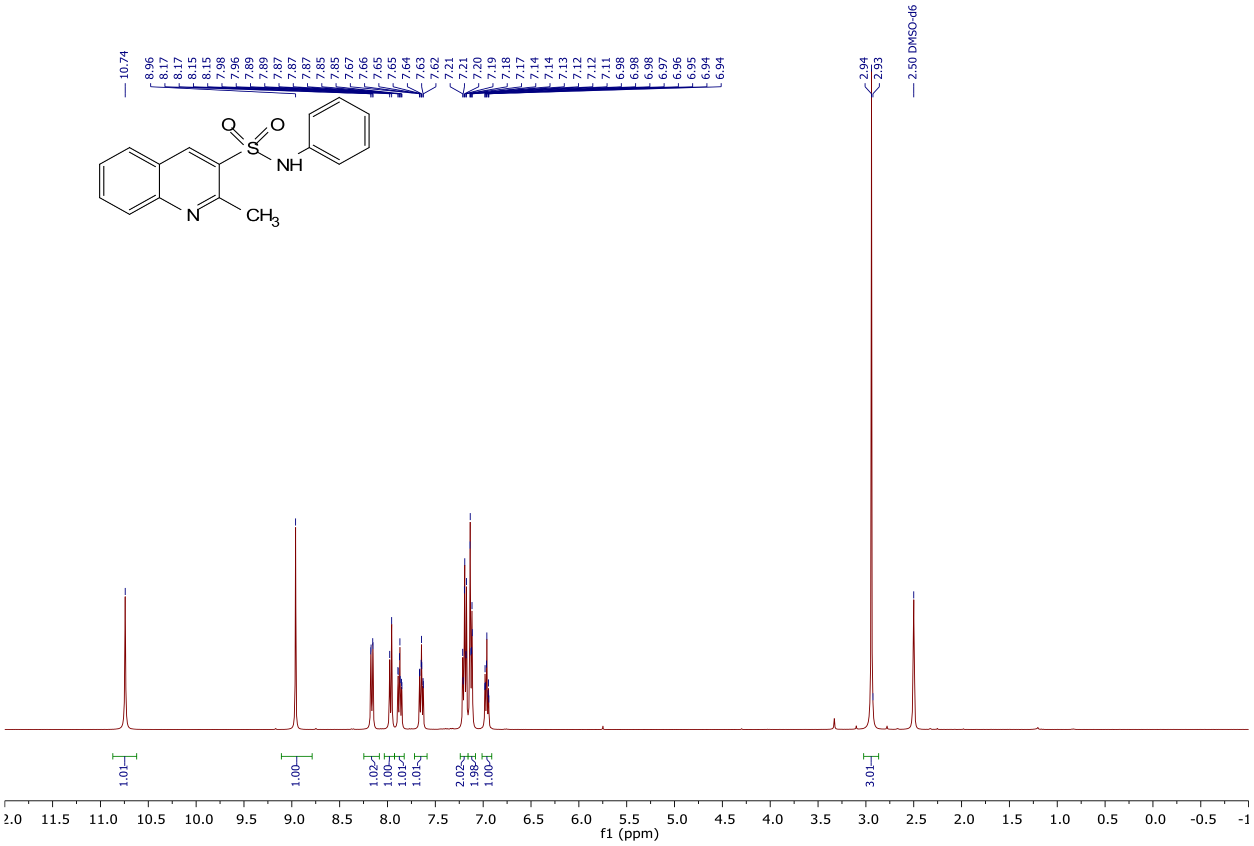

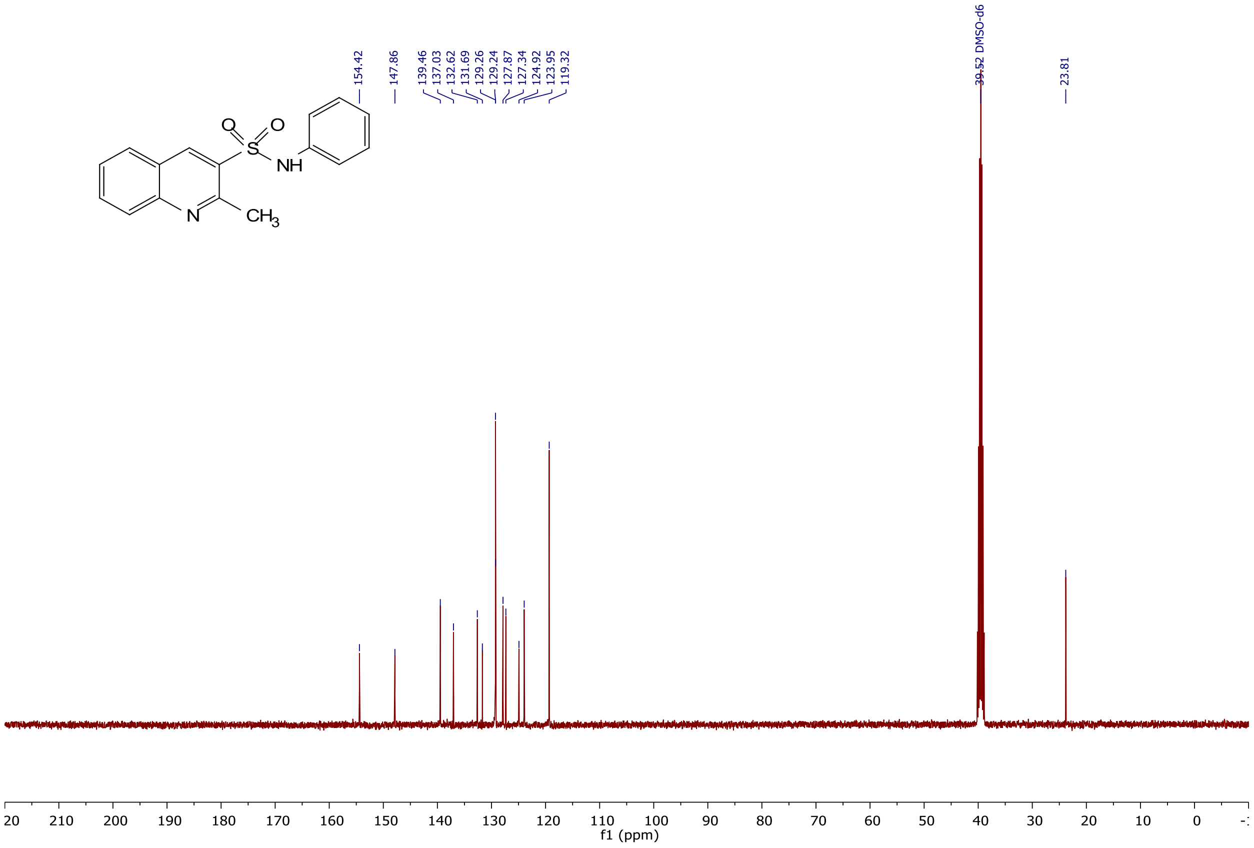

Copies of ^{1}H (400.13 MHz, DMSO-d_6) and $^{13}C\{^{1}H\}$ (100.61 MHz, DMSO-d_6) of **5j**

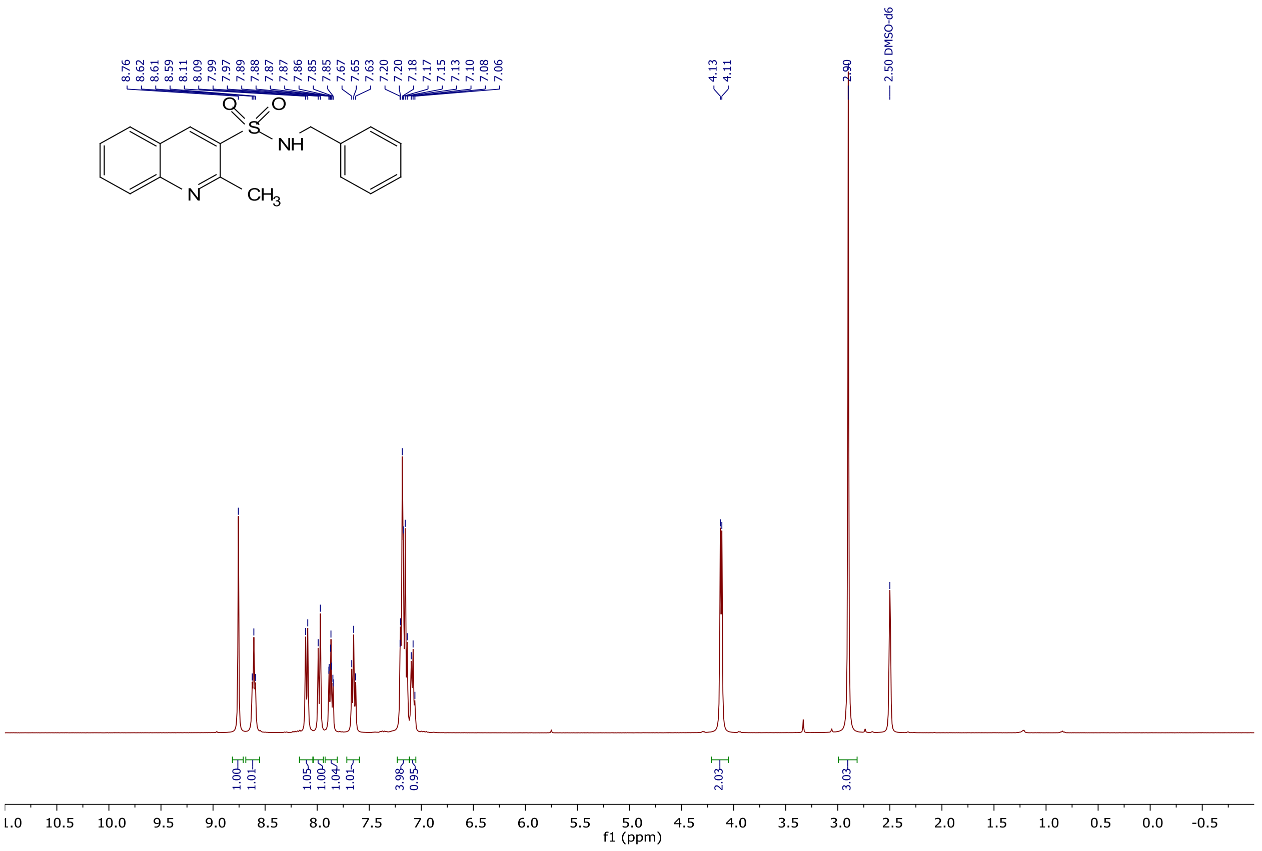

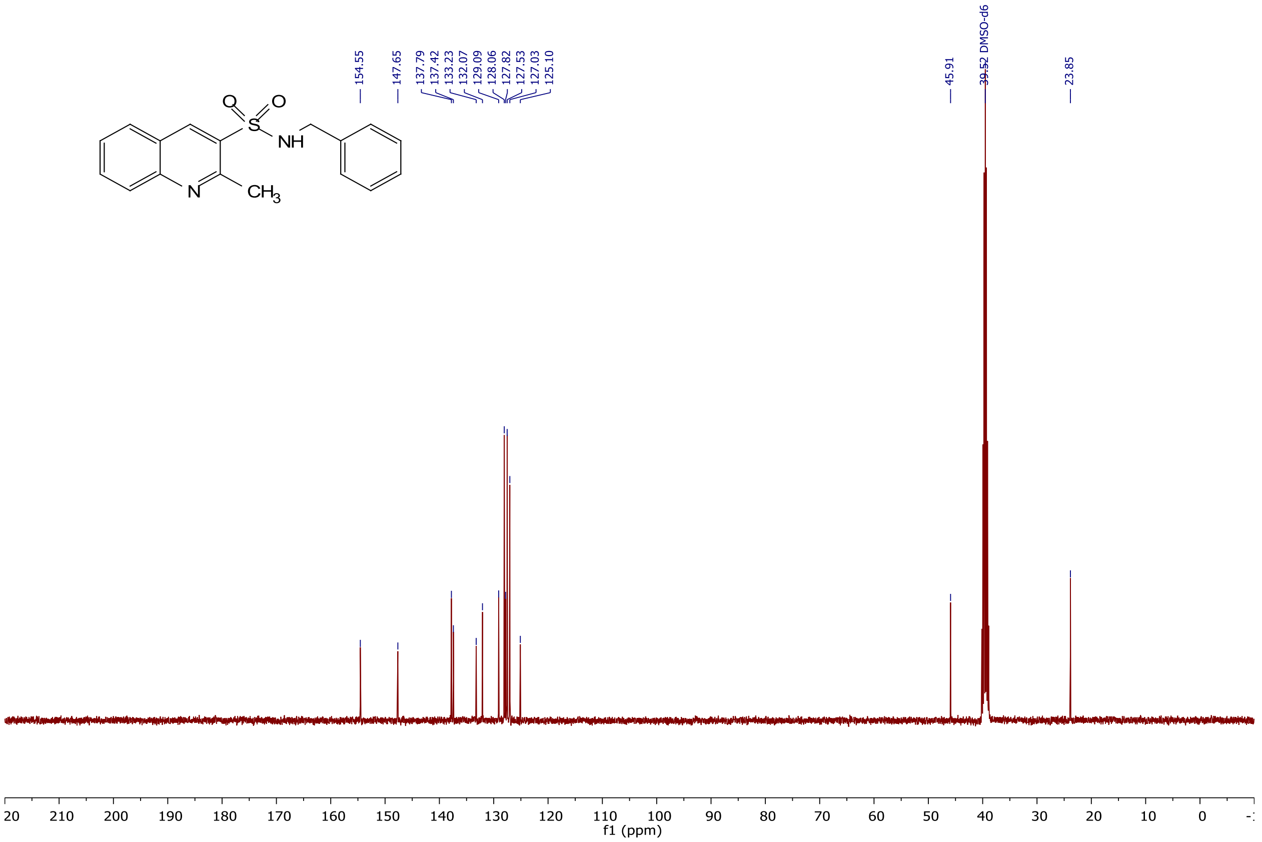

Copies of ^{1}H (400.13 MHz, $CDCl_3$) and $^{13}C\{^{1}H\}$ (100.61 MHz, $CDCl_3$) of **5k**

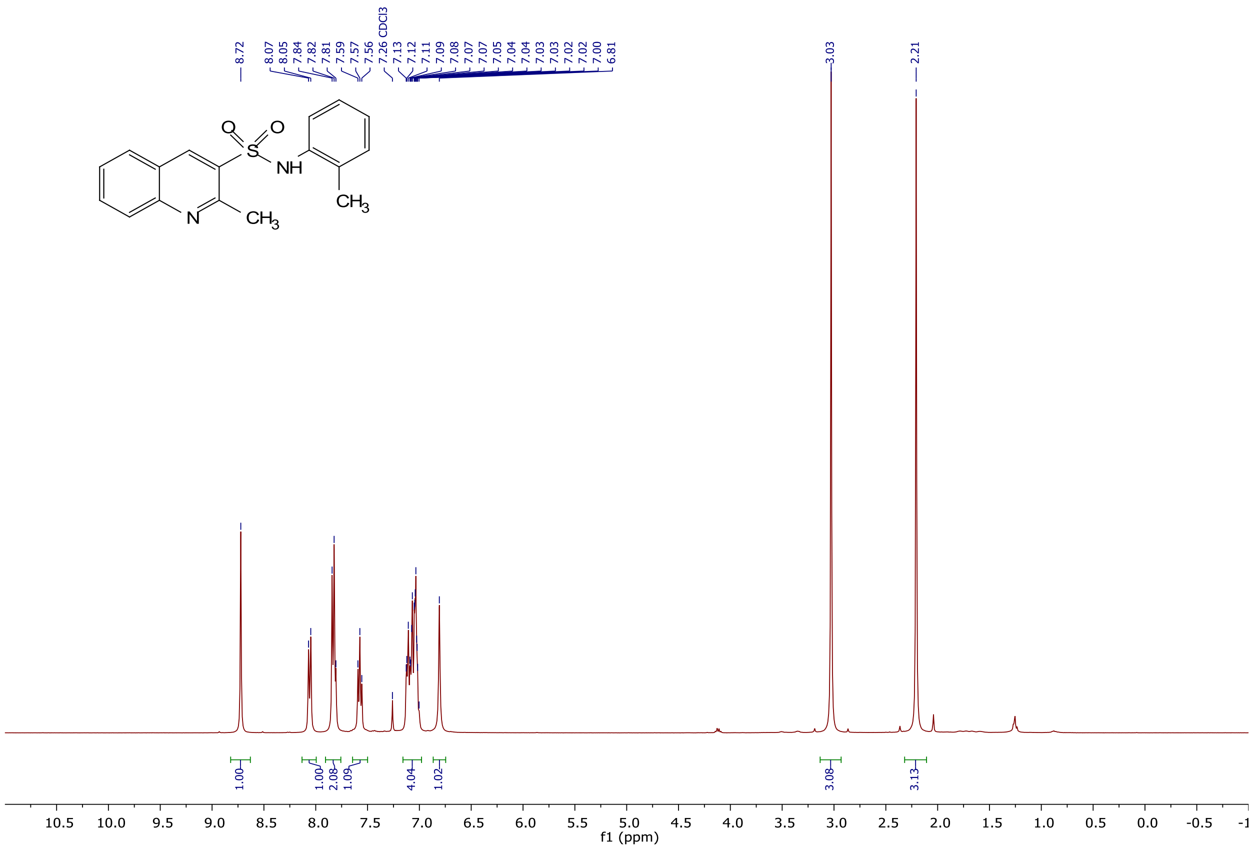

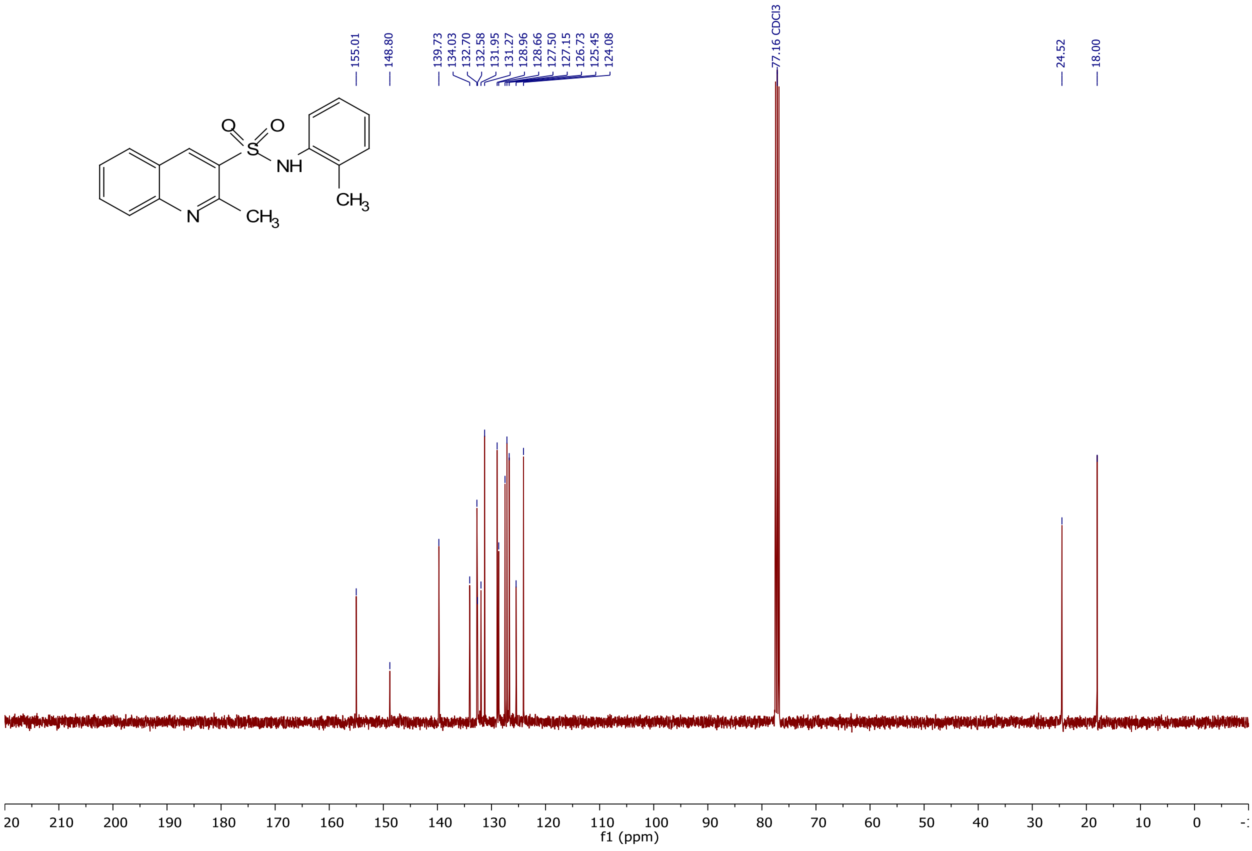

Copies of ^{1}H (400.13 MHz, DMSO-d_6) and $^{13}C\{^{1}H\}$ (100.61 MHz, DMSO-d_6) of **5l**

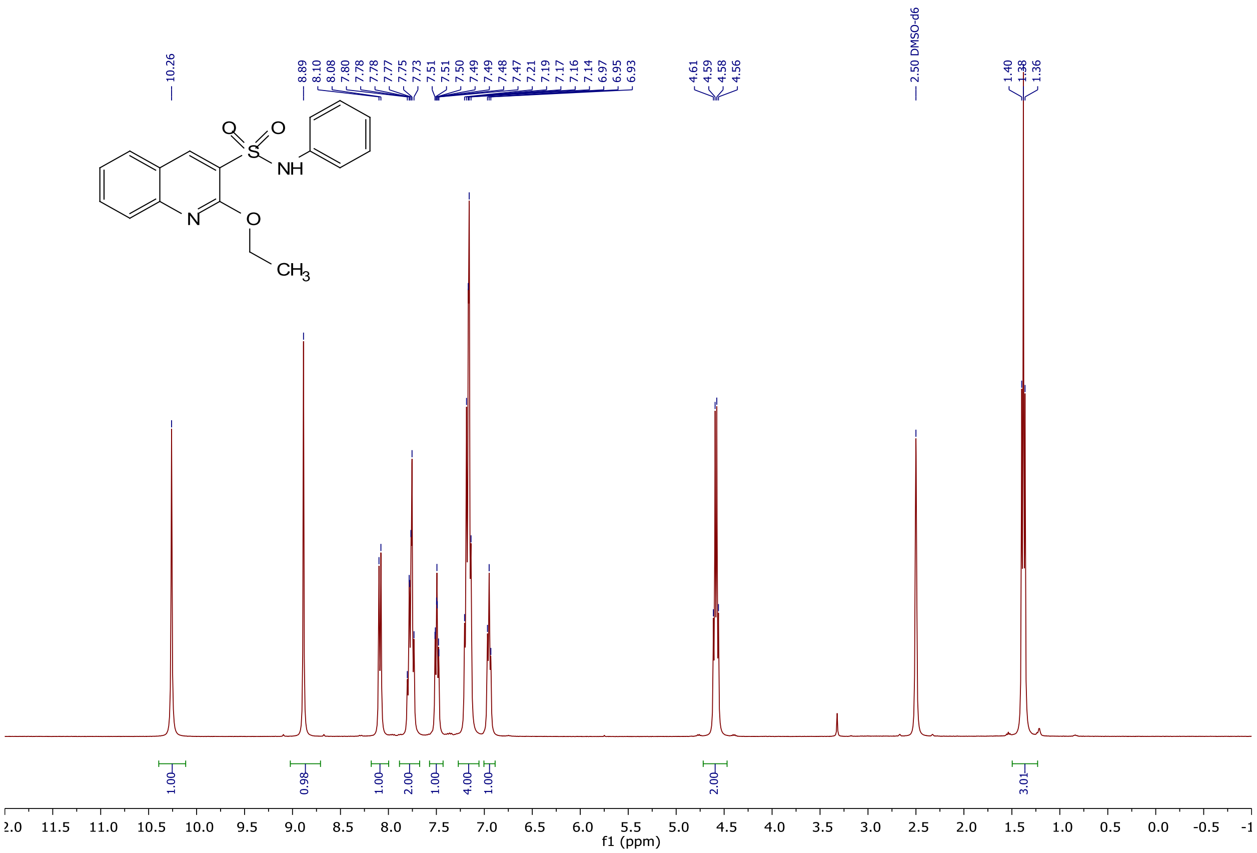

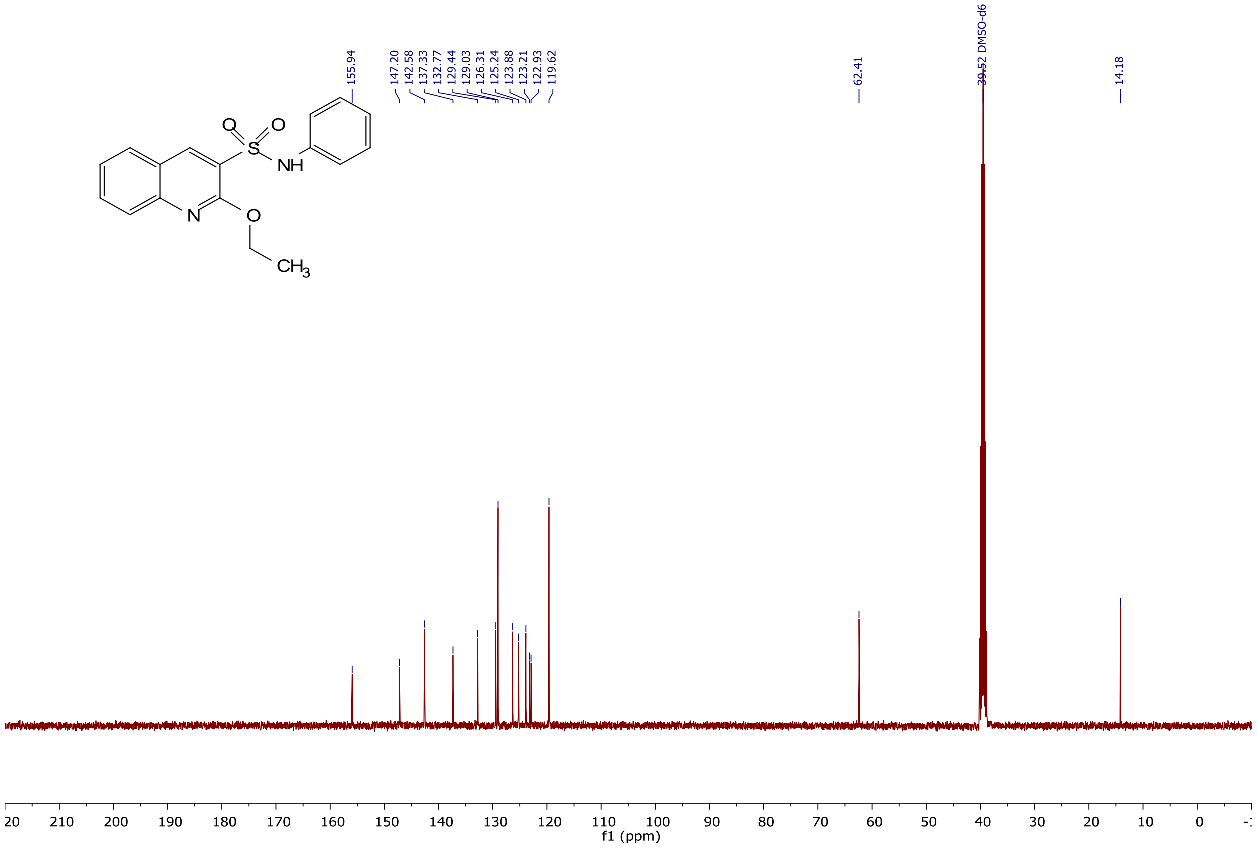

Copies of ^{1}H (400.13 MHz, $CDCl_3$) and $^{13}C\{^{1}H\}$ (100.61 MHz, $CDCl_3$) of **5m**

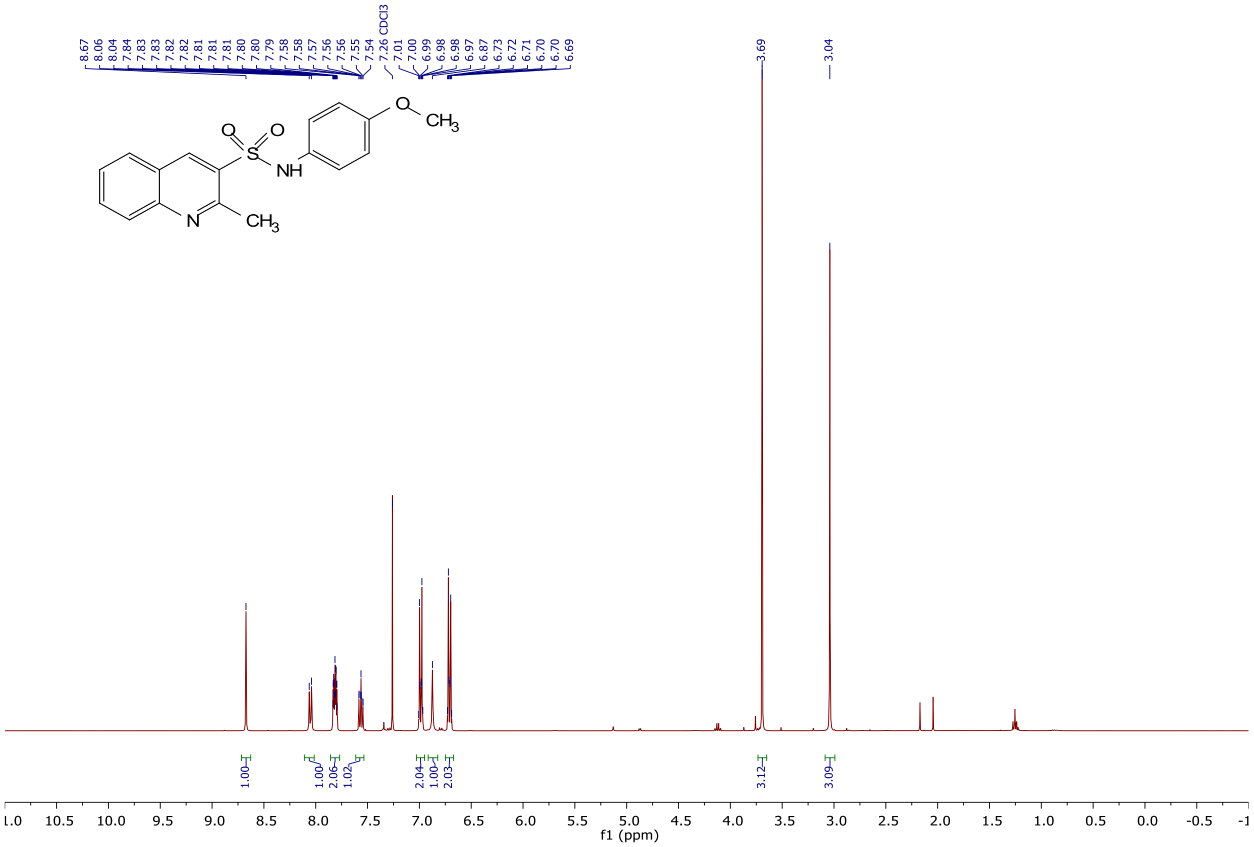

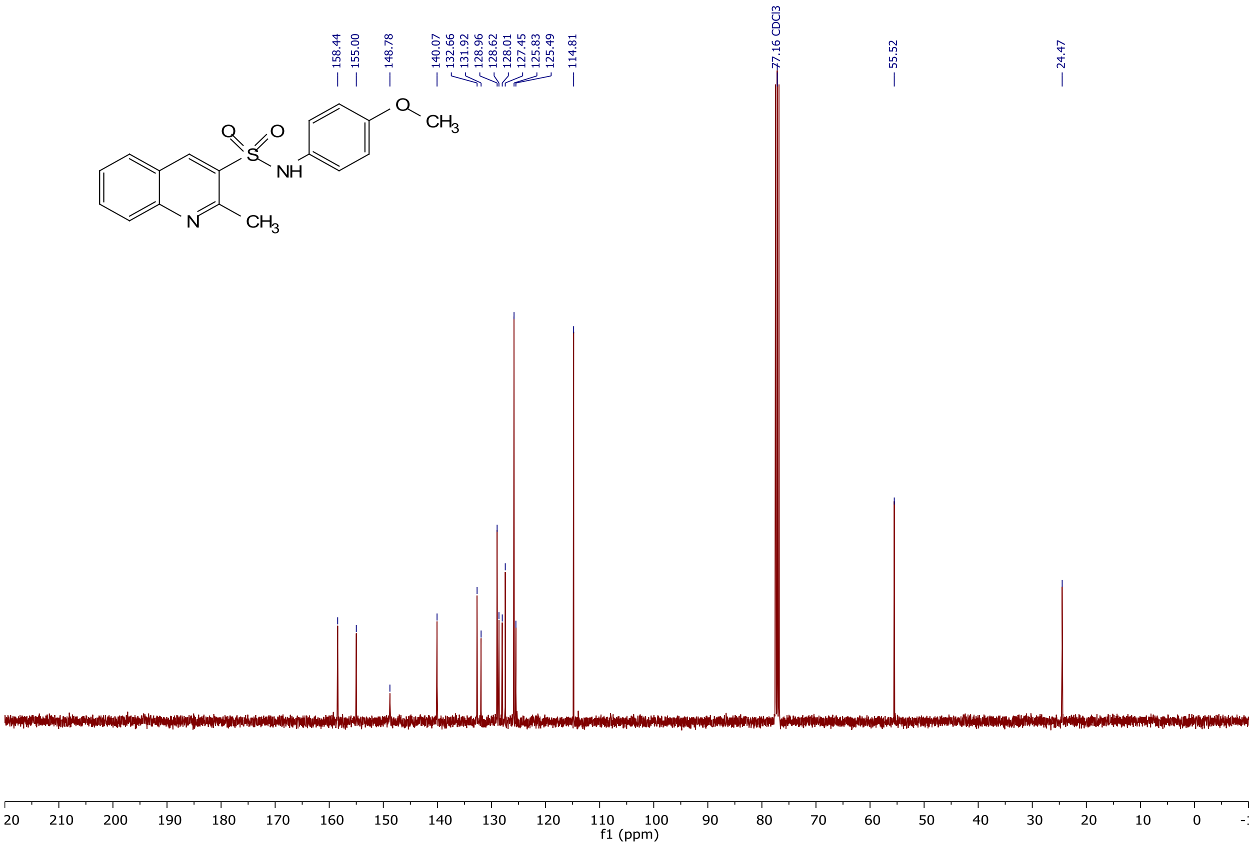

Copies of ^{1}H (400.13 MHz, $CDCl_3$) and $^{13}C\{^{1}H\}$ (100.61 MHz, $CDCl_3$) of **5n**

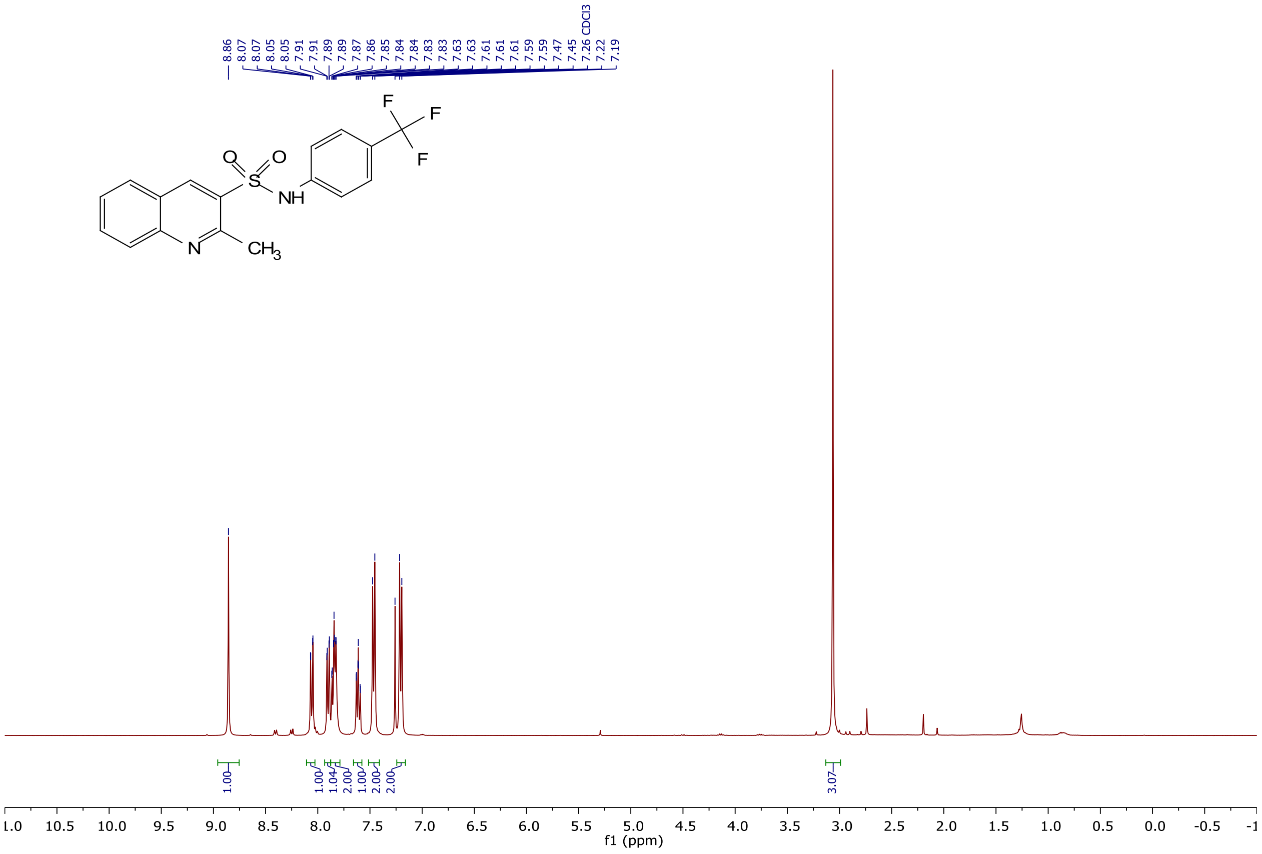

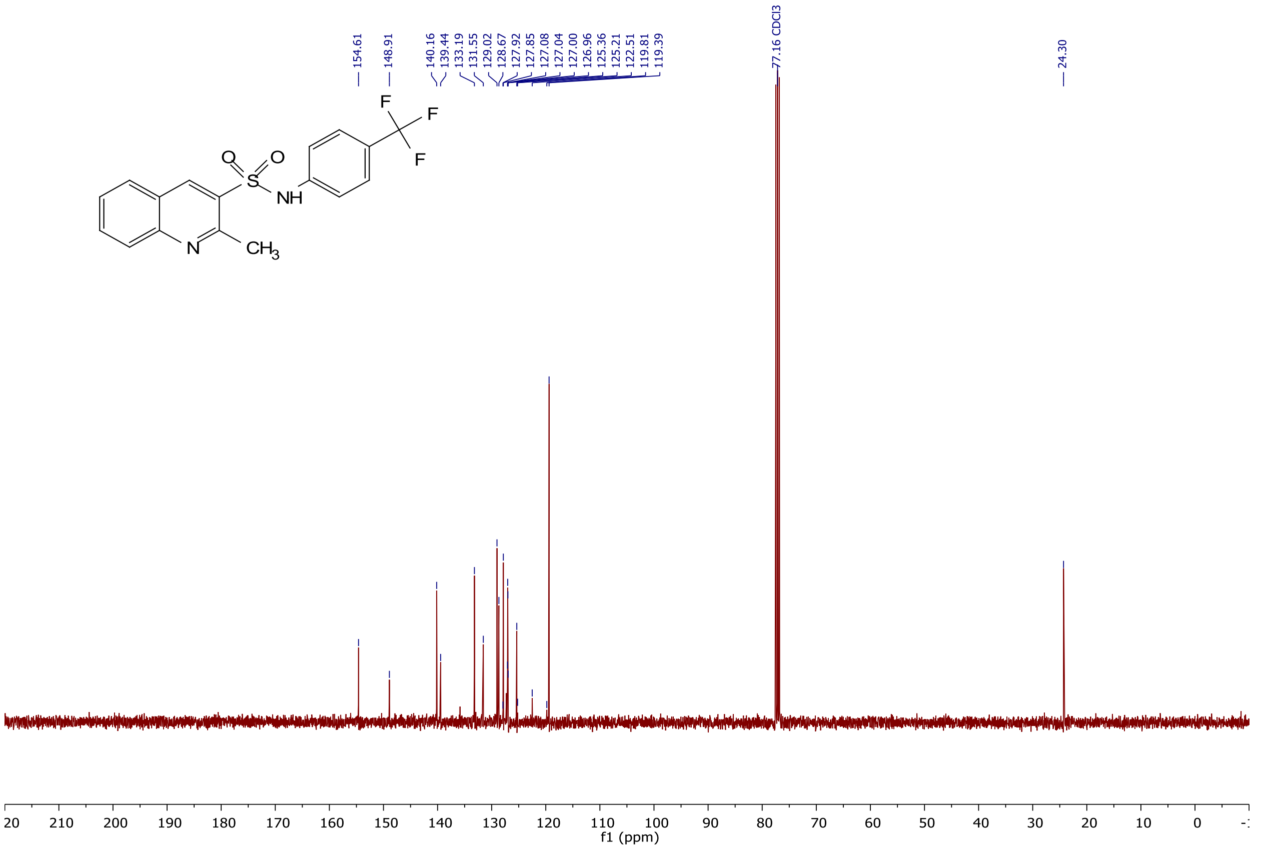

Copy of $^{19}F\{^{1}H\}$ (376.50 MHz, $CDCl_3$) spectrum of **5n**

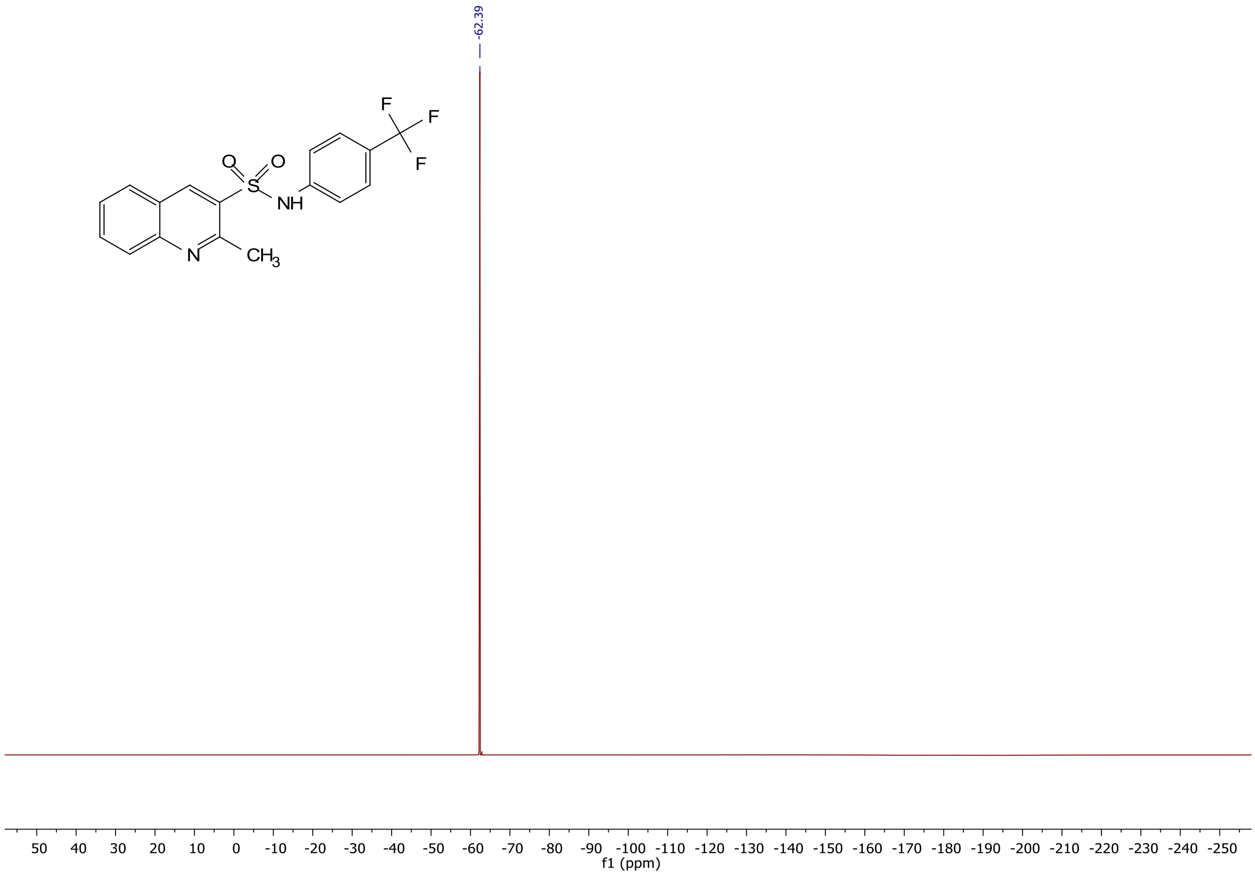

Copies of ^{1}H (400.13 MHz, $CDCl_3$) and ^{13}C{^{1}H} (100.61 MHz, $CDCl_3$) of **5o**

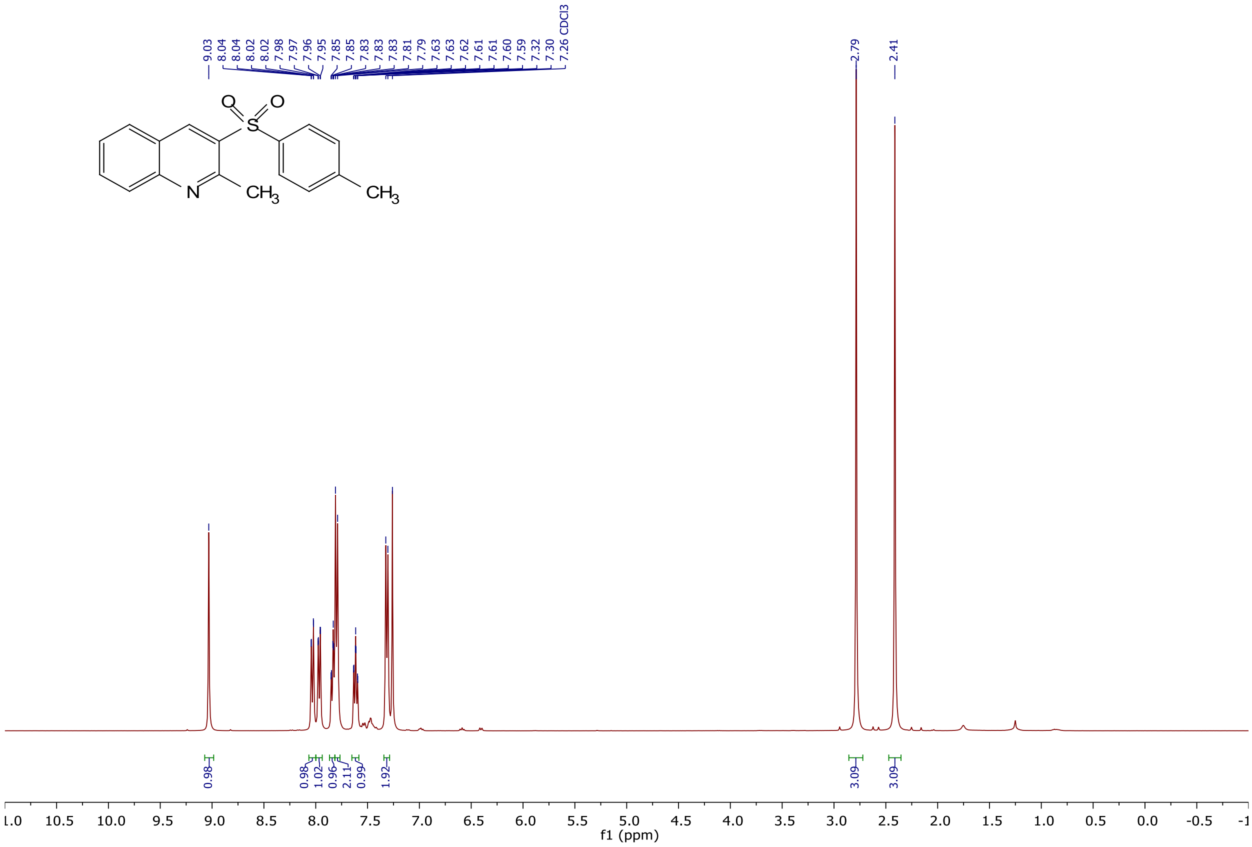

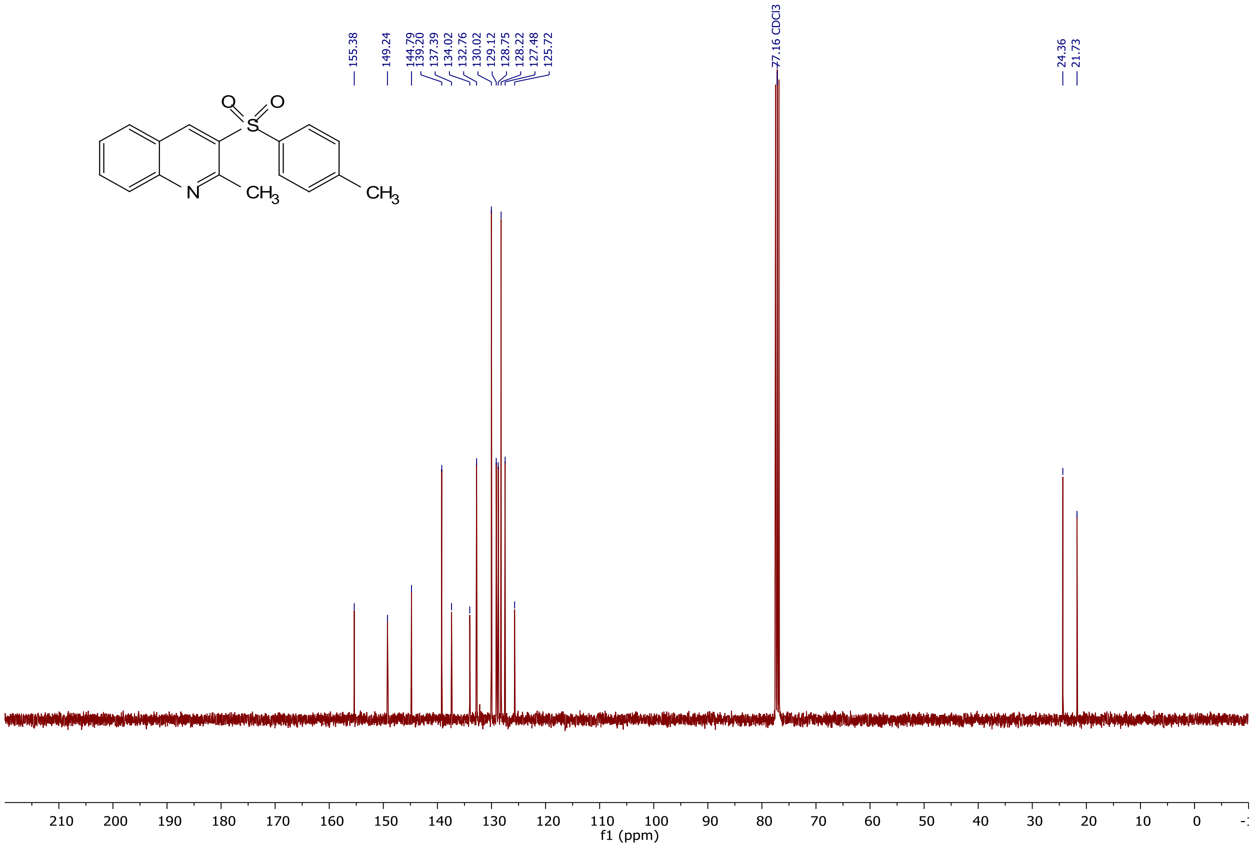

Copies of ^{1}H (400.13 MHz, $CDCl_3$) and $^{13}C\{^{1}H\}$ (100.61 MHz, $CDCl_3$) of **5p**

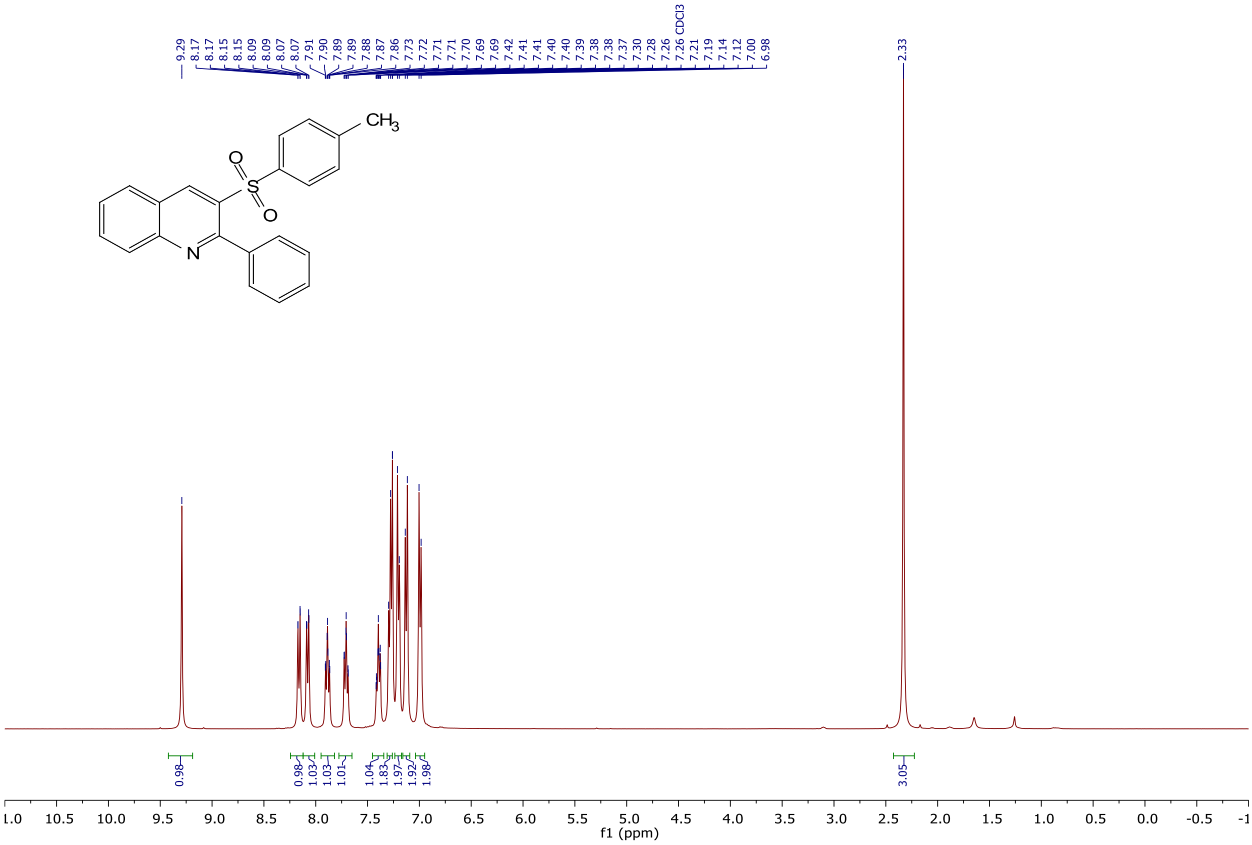

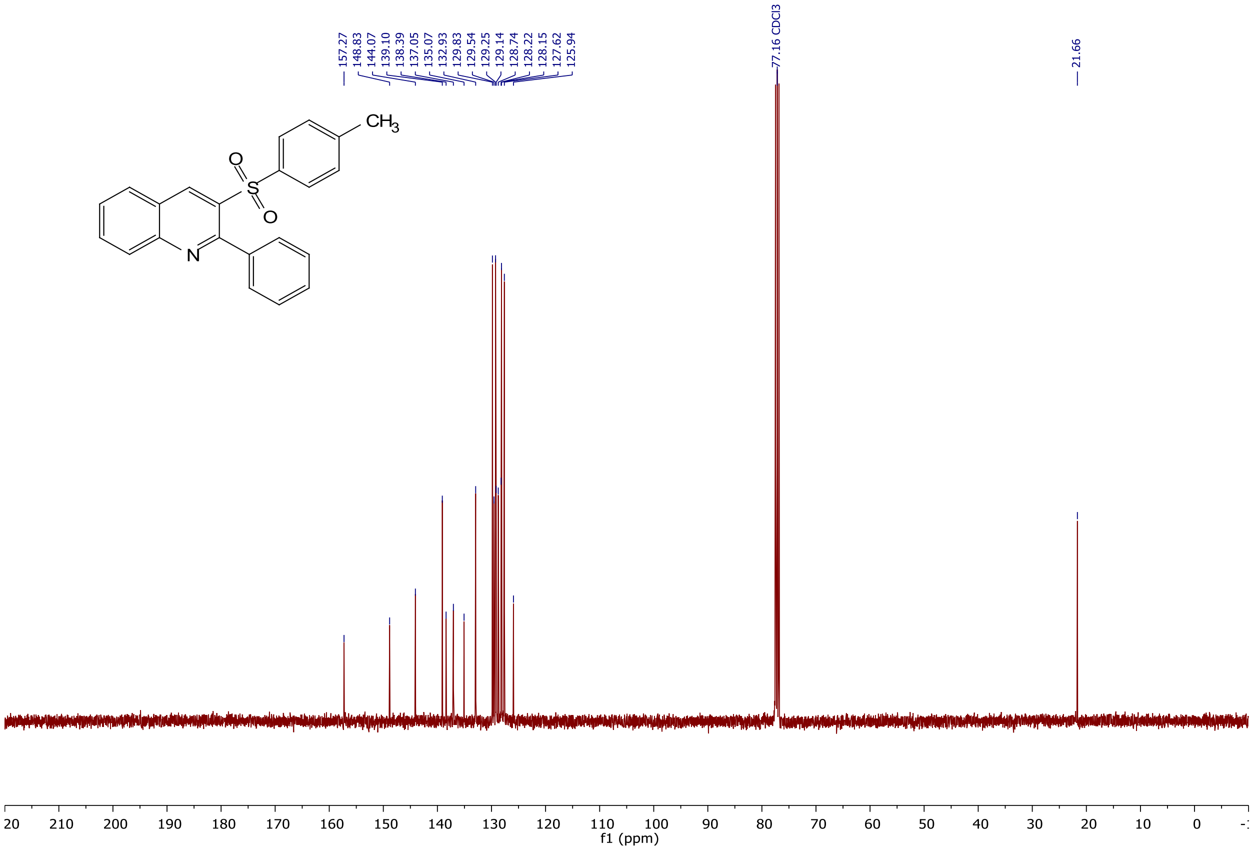

Copies of ^{1}H (400.13 MHz, $CDCl_3$) and $^{13}C\{^{1}H\}$ (100.61 MHz, $CDCl_3$) of **5q**

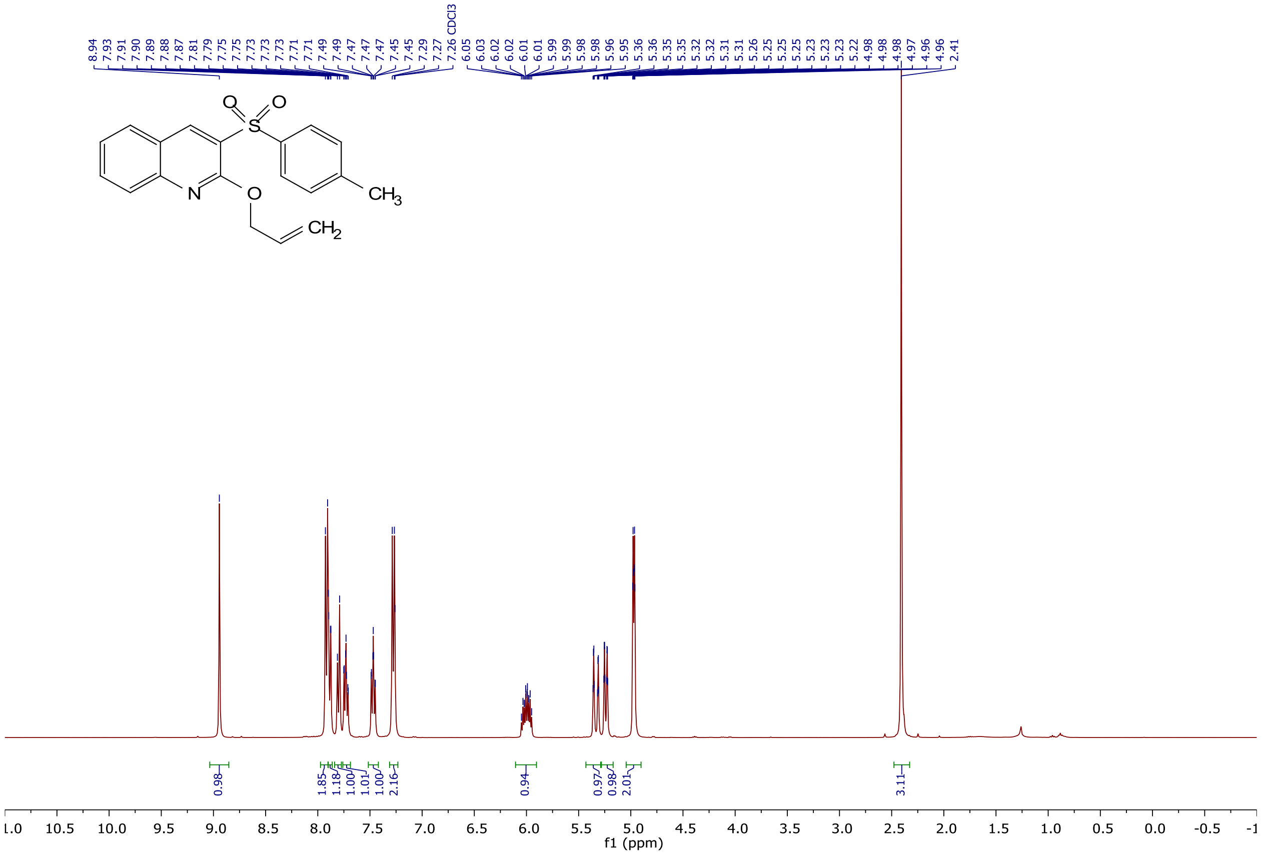

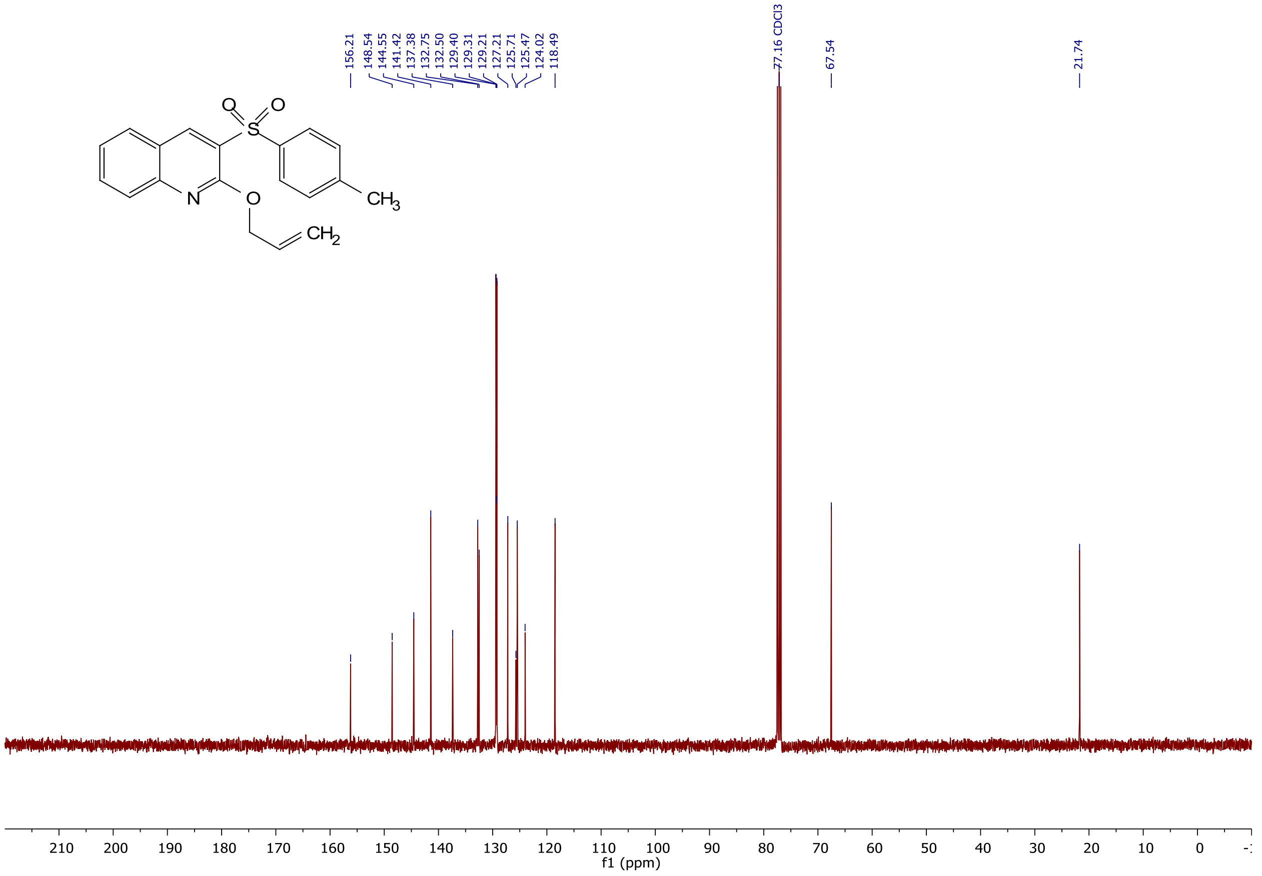

VI. X-ray crystallographic data

Crystallographic data for compound **5a**. X-ray Single Crystal Analysis was performed on a SuperNova diffractometer. Crystal was kept at 100(2) K during data collection. Using Olex2 [11], the structure was solved with the SHELXT [12] structure solution program using Intrinsic Phasing and refined with the SHELXL [13] refinement package using Least Squares minimization.

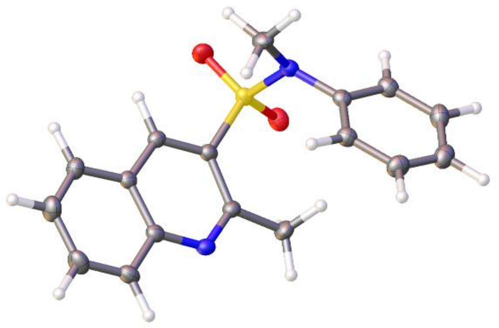

Figure S1. ORTEP representation of compound **5a** (thermal ellipsoids are shown at 50% probability).

Table S1. Crystal data and structure refinement for **5a**

**CCDC**	**2242072**
Empirical formula	$C_{17}H_{16}N_2O_2S$
Formula weight	312.38
Temperature/K	100(2)
Crystal system	monoclinic
Space group	$P2_1/n$
a/Å	8.3096(2)
b/Å	5.71690(10)
c/Å	31.2551(6)
α/°	90
β/°	93.640(2)
γ/°	90
Volume/Å^3	1481.78(5)
Z	4
ρ_{calc}g/cm^3	1.4
μ/mm^{-1}	2.015
F(000)	656
Crystal size/mm^3	0.4 × 0.06 × 0.02
Radiation	Cu Kα (λ = 1.54184)
2Θ range for data collection/°	5.666 to 151.92
Index ranges	$-9 \leq h \leq 10$, $-6 \leq k \leq 7$, $-39 \leq l \leq 39$
Reflections collected	8474
Independent reflections	2941 [R_{int} = 0.0469, R_{sigma} = 0.0414]
Data/restraints/parameters	2941/0/201
Goodness-of-fit on F^2	1.055
Final R indexes [I>=2σ (I)]	R_1 = 0.0472, wR_2 = 0.1283
Final R indexes [all data]	R_1 = 0.0529, wR_2 = 0.1316
Largest diff. peak/hole / e Å^{-3}	0.33/-0.55

VII. References

1. Malkova, K.; Bubyrev, A.; Krivovicheva, V.; Dar'in, D.; Bunev, A.; Krasavin, M. *Beilstein J. Org. Chem.* **2022,** *18*, 1636-1641.
2. Sabatié, A.; Végh, D.; Loupy, A.; Floch, L. u. *Arkivoc* **2001,** *2001* (6), 122-128.
3. Becher, J.; Begtrup, M.; Gjerløv, A.; Larsen, S.; Dehaen, W.; Christensen, L.; Napoli, A.; Sindona, G.; Aksnes, D.; Francis, G.; Aaberg, A. *Acta Chem. Scand.* **1995,** *49*, 57-63.
4. Bubyrev, A.; Dar'in, D.; Kantin, G.; Krasavin, M. *Eur. J. Org. Chem.* **2020,** *2020* (27), 4112-4115.
5. Klochkova, A.; Bubyrev, A.; Dar’in, D.; Bakulina, O.; Krasavin, M.; Sokolov, V. *Synthesis* **2021,** *53* (10), 1795-1804.
6. Swenson, R. E.; Sowin, T. J.; Zhang, H. Q. *J. Org. Chem.* **2002,** *67* (26), 9182-9185.
7. Bubyrev, A.; Malkova, K.; Kantin, G.; Dar’in, D.; Krasavin, M. *J. Org. Chem.* **2021,** *86* (23), 17516-17522.
8. Chen, C.-C.; Ho, J.-C.; Chang, N.-C. *Tetrahedron* **2008,** *64* (45), 10350-10354.
9. Ma, H.; Liu, S.; Zhu, S.; Bi, W.; Chen, X.; Zhao, Y. *Phosphorus Sulfur Silicon Relat. Elem.* **2017,** *192* (8), 887-895.
10. Wang, F.; Xu, P.; Wang, S.-Y.; Ji, S.-J. *Org. Lett.* **2018,** *20* (8), 2204-2207.
11. Dolomanov, O. V.; Bourhis, L. J.; Gildea, R. J.; Howard, J. A. K.; Puschmann, H. *J. Appl. Crystallogr.* **2009,** *42* (2), 339-341.
12. Sheldrick, G. *Acta Crystallogr. A* **2015,** *71* (1), 3-8.
13. Sheldrick, G. *Acta Crystallogr. C* **2015,** *71* (1), 3-8.
